# Unraveling the stress response and biosorption mechanisms of *Aspergillus niger* to rare earth element cerium(III) based on transcriptomics and DNA methylomics

**DOI:** 10.3389/fmicb.2025.1674444

**Published:** 2025-10-27

**Authors:** Jingqi Liu, Donghua Tan, Huangfeng Qiu, Yuting Liang, Haiyan Wu, Yu Yang, Hongbo Zhao

**Affiliations:** ^1^School of Minerals Processing and Bioengineering, Central South University, Changsha, Hunan, China; ^2^Key Laboratory of Biohydrometallurgy of Ministry of Education, Central South University, Changsha, Hunan, China; ^3^Hunan Provincial Engineering Research Center for Mineral and Biological Metallurgy, Central South University, Changsha, Hunan, China

**Keywords:** *Aspergillus niger*, REEs adsorption, REEs tolerance mechanisms, metabolic adaptation, transcriptomics, DNA methylation, bioleaching applications

## Abstract

Rare earth elements (REEs) represent critical industrial resources, yet conventional extraction methods face substantial environmental and efficiency constraints. Fungal bioleaching emerges as an eco-friendly alternative, leveraging organic acid secretion to facilitate REEs dissolution and adsorption. However, progressive REEs accumulation inhibits microbial activity, with fungal resistance mechanisms remaining incompletely understood. Here, we report the discovery of *Aspergillus niger* FH1, a highly REEs-tolerant strain exhibiting remarkable Ce(III) tolerance (600 mg/L maximum) and achieving 74.05% adsorption efficiency under optimized conditions. Integrated physicochemical characterization (SEM, FTIR, XPS) revealed dual adsorption mechanisms: physical entrapment evidenced by Ce(III)-induced cellular invagination, and chemical monolayer binding via extracellular functional group coordination (amino, hydroxyl, carboxyl, carbonyl, phosphate), with specific moieties enabling Ce(III) capture through surface complexation. Transcriptomic analysis identified 3,733 differentially expressed genes under Ce(III) stress. Functional annotation (GO/KEGG) demonstrated: (1) Significant repression of oxidative phosphorylation genes; (2) Concomitant upregulation of glycolysis, pentose phosphate pathway, and amino acid metabolism genes indicating metabolic rerouting for energy maintenance; (3) Enhanced expression of antioxidative/chelating metabolite synthesis pathways. Whole-genome bisulfite sequencing revealed conserved global 5mC DNA methylation levels (0.32% vs. 0.36% in controls) with preferential CHH-context targeting. Collectively, these adaptation strategy combines extracellular sequestration, metabolic plasticity, and stress mitigation to confers exceptional resilience against rare earth metal toxicity. The demonstrated adsorption-tolerance synergy positions *A. niger* FH1 as an important bioagent for sustainable recovery of recalcitrant rare earth resources.

## Introduction

Rare earth elements, which are often referred to as “industrial vitamins,” possess distinctive optical, chemical, and magnetic properties (Charlotte Maluleke et al., 2023; Zhang et al., 2023). These critical industrial resources are extensively applied in fields including industrial catalysis, optics, electronics and battery technology, pharmaceuticals, and superconductivity (Zhang et al., 2023), driving immense demand. However, the scarcity and non-renewable nature of REEs pose significant challenges. Conventional extraction and recovery techniques for REEs, encompassing electrochemical methods, ion exchange, membrane separation, and solvent extraction, are characterized by inherent limitations. These techniques are associated with operational complexity, substantial expenses, and the potential for secondary pollution. During the mining process, a significant volume of wastewater is produced, containing substantial quantities of REEs. This has resulted in substantial resource wastage and environmental contamination (Fan et al., 2021). In contrast, microorganisms have the capacity to adsorb REEs through functional groups located on their cell surfaces. This process can occur via mechanisms such as electrostatic attraction, ion exchange, and surface complexation (Andrès and Gérente, 2011; Brown et al., 2023; Panda et al., 2021; Shin et al., 2015; Wang L. et al., 2017). This method of REEs separation and recovery has been shown to be both environmentally friendly and efficient (Meng et al., 2022).

While microbe-mediated adsorption provides a green route for REE enrichment, its efficiency is influenced by multiple variables including adsorption time, biomass dosage, pH, temperature, and initial ion concentration. Research by Maleke et al. (2019) demonstrated that *Thermus scotoductus* can specifically adsorb Eu^3 +^ via electrostatic attraction using functional groups such as PO3-4 and -COOH on its cell surface. Furthermore, studies indicate that different functional groups exhibit varying adsorption capacities for different rare earth ions: PO3-4 shows stronger adsorption for light REEs, while both PO3-4 and -COOH exhibit similar adsorption capacities for medium and heavy REEs (Martinez et al., 2014). According to Takahashi et al. (2005), *Escherichia coli* and *Bacillus subtilis* differ in the types and efficiency of REEs adsorption, attributable to variations in the types and quantities of surface functional groups. Adsorption efficiency is also pH-dependent: light REEs are better adsorbed at pH >4, while medium and heavy REEs are strongly adsorbed at pH <4 (Martinez et al., 2014). Beyond bacteria, fungi have also been reported to adsorb rare earth ions. Giese et al. found that the fungus *Botryosphaeria rhodina*, used in β-glucan production, can adsorb La(III) and Sm(III). Its adsorption rate decreased from 100 to 25% as the initial REEs concentration increased from 15 mg/L to 100 mg/L (Giese et al., 2019). Zinicovscaia et al. similarly demonstrated that *Saccharomyces cerevisiae* can adsorb Dy(III) from wastewater at pH 3.0, reaching adsorption equilibrium within 1 h with a maximum capacity of 5.84 mg/g (Zinicovscaia et al., 2009). Bergsten et al. also found that various fungi exhibit high resistance to REEs toxicity and can survive in REEs-rich environments (Bergsten-Torralba et al., 2020).

However, high concentrations of REEs ions in the adsorption environment can exert toxic effects on microorganisms, inhibiting their growth and thereby reducing REEs recovery efficiency (Gadd, 1994). For instance, Ce(III) exposure can induce oxidative stress, cellular damage, and toxicity in aquatic organisms (Malhotra et al., 2020; Pulido-Reyes et al., 2015; Revel et al., 2025). Studies suggest that enhancing the tolerance of leaching strains can improve the leaching efficiency of target metals. Shah et al. reported that using Al-tolerant strains adapted through domestication significantly increased the leaching efficiency of low-grade bauxite to 97% compared to wild strains (Shah et al., 2020). Moreover, isolated tolerant strains often possess inherent adsorption capacity for target metals. Wang et al. found that *Penicillium* sp. ZD28, isolated from rare earth mining soil, could grow in medium containing 800 mg/L Y(III), demonstrating high tolerance. Adsorption assays revealed an adsorption rate of up to 99% for Y(III) when grown in environments with concentrations below 600 μM (Wang W. et al., 2020).

Collectively, these findings underscore fungi as significant microbial resources for REE adsorption applications. However, substantial knowledge gaps remain regarding the underlying mechanisms and optimization of fungal REE adsorption. To address this, our study isolated a novel fungal strain exhibiting high tolerance to the representative REE ion Ce(III). We systematically investigated its tolerance profile and adsorption performance under varying conditions (pH, initial Ce(III) concentration, biomass dosage). Adsorption kinetics and isotherms were determined, while adsorption mechanisms were elucidated using X-ray photoelectron spectroscopy (XPS) and Fourier transform infrared spectroscopy (FTIR) characterization. Furthermore, we employed RNA sequencing (RNA-Seq) to analyze transcriptomic responses of the *A. niger* FH1 strain to Ce(III) stress, validating key findings via real-time quantitative PCR (RT-qPCR). Functional enrichment analysis of differentially expressed genes identified pathways central to Ce(III) resistance. Complementarily, whole-genome bisulfite sequencing (WGBS) was utilized to profile genome-wide DNA methylation dynamics in *A. niger* FH1 under Ce(III) exposure.

## Materials and methods

### Fungal strain and growth conditions

The *A. niger* strain FH1, which was isolated and preserved in this study, originated from soil samples collected in a rare-earth mining area in Inner Mongolia Province, China. Four distinct media were utilized: a Screening Medium consisting of 10 g/L NaCl, 10 g/L tryptone, 5 g/L yeast extract, and 15 g/L agar, supplemented with filter-sterilized (0.22 μm) CeCl3 stock solution after autoclaving (121 °C, 20 min) to a final Ce(III) concentration of 50 mg/L; Potato Dextrose Agar (PDA) containing 6 g/L potato starch, 20 g/L glucose, and 15 g/L agar, sterilized at 115 °C for 30 min; Yeast Extract-Peptone-Dextrose (YPD) medium composed of 20 g/L glucose, 20 g/L tryptone, 10 g/L yeast extract, and 15 g/L agar (for solid medium), also sterilized at 115 °C for 30 min; and a Sphere-Forming Medium with 10 g/L glucose, 2 g/L NH_4_Cl, 2 g/L KH_2_PO_4_, 0.5 g/L MgSO_4_, and 2 g/L yeast extract, sterilized under the same conditions.

All liquid cultures were incubated at 30 °C with shaking (180 rpm). Experiments were performed in triplicate unless otherwise stated.

### Screening and identification of Ce(III)-tolerant strain

Soil suspensions were prepared by vortexing 0.5 g of cryopreserved soil in 5 mL sterile saline. Serial dilutions (10^3^ and 10^4^) were plated on Ce(III)-supplemented screening medium. Colonies exhibiting Ce(III) tolerance were purified on PDA plates and designated as strain FH1.

For spore preparation, FH1 spores were harvested using 0.1% Tween 80, washed with sterile deionized water, and adjusted to OD_270_ = 0.1. Spore suspensions were stored at 4 °C until use (Ma et al., 2023). Genomic DNA was extracted from log-phase hyphae (YPD-grown) using the DE241 Fungal DNA Kit (Coolaber, China). The ITS region was amplified using primers ITS1 (5′-TCCGTAGGTGAACCTGCGG-3′) and ITS4 (5′-TCCTCCGCTTATTGATATGC-3′), with the PCR products sequenced by Tsingke Biotechnology (China). For sequence identification, the obtained ITS sequence was queried against the NCBI GenBank database^1^ using BLASTn (Basic Local Alignment Search Tool for nucleotide sequences) with default parameters: an E-value threshold of 1e-5, match/mismatch scores of +2/−3, and a gap penalty of 5 (existence) and 2 (extension). From the BLAST results, we selected the top-hit sequences based on criteria of ≥97% sequence similarity and ≥90% query coverage, prioritizing sequences from type strains or taxonomically validated isolates to ensure phylogenetic relevance. Phylogenetic analysis was then performed using MEGA12, incorporating these selected reference sequences.

### Ce(III) tolerance and batch adsorption assays

#### Tolerance assay

FH1 spores (1% v/v) were inoculated into YPD medium containing 0–700 mg/L Ce(III). Biomass (dry weight, drying at 80 °C until the weight of the organisms no longer changes) and pH were measured daily for 6 days.

#### Adsorption optimization

FH1 hyphae obtained from sphere-forming medium were harvested, washed, and resuspended in physiological saline solution, after which the adsorption efficiency (A_e_, %) and capacity (q_e_, mg/g) were systematically evaluated under a range of operating conditions, including pH values from 3.0 to 7.0, initial Ce(III) concentrations between 40 and 120 mg/L, and biomass dosages ranging from 500 to 2500 mg/L.

Pre- and post-adsorption samples were filtered through 0.22 μm membranes, and Ce(III) concentrations were quantified via inductively coupled plasma optical emission spectrometry (ICP-OES). The adsorption efficiency (*A*_*e*_, %) and adsorption capacity (*q*_*e*_, mg/g) of strain FH1 were calculated by Equations 1, 2, respectively.


(1) $$A_{e}=\frac{C_{0}-C_{t}}{C_{0}}\times 100\%$$

(2) $$q_{e}=\frac{\left(C_{0}-C_{t}\right)\,V}{M}$$

Where: *C*_0_ (mg/L) and *C*_*t*_ (mg/L) represent the initial and equilibrium concentrations of the target rare earth ion Ce(III) before and after adsorption, respectively. *M* (g) denotes the dry biomass weight used for adsorption, and *V* (L) is the total volume of the adsorption solution.

#### Kinetic and isotherm modeling

In order to describe and predict the process of adsorption of rare earth ions more accurately, The experimental data of Ce (III) adsorption by *A.niger* FH 1 strain were fitted by using typical quasi-first-order (Equation 3) and quasi-second-order kinetic model (Equation 4). In addition, to explore the relationship between the adsorption amount at equilibrium and the initial Ce (III) concentration in the adsorption process, The two most widely used isothermal adsorption models, Freundlich model (Equation 5) and Langmuir model (Equation 6), were used to fit the adsorption data.


(3) $$\mathrm{Pseudo}-\mathrm{first}-\mathrm{order}:\text{ln}\,\left(q_{\text{e}}-q_{t}\right)=\text{ln}\,q_{\text{e}}-k_{1}\,t$$

(4) $$\mathrm{Pseudo}-\mathrm{second}-\mathrm{order}:\frac{t}{q_{t}}=\frac{1}{k_{2}\,q_{\text{e}}^{2}}+\frac{t}{q_{\text{e}}}$$

(5) $$\mathrm{Freundlich}\,\mathrm{model}:q_{\text{e}}=K_{\text{F}}\,C_{\text{e}}^{1/n}$$

(6) $$\mathrm{Langmuir}\,\mathrm{model}:q_{\text{e}}=\frac{q_{\text{m}}\,K_{\text{L}}\,C_{\text{e}}}{1+K_{\text{L}}\,C_{\text{e}}}$$

Where: *q*_*t*_ (mg/g) denotes the adsorption capacity of *A. niger* FH1 for Ce(III) at time t; *k*_1_ (min^−1^) and *k*_2_ (mg^⋅^g^−1⋅^min^−1^) represent the pseudo-first-order and pseudo-second-order rate constants, respectively; *C*_*e*_ (mg/L) is the equilibrium concentration of Ce(III) after adsorption; *q*_*m*_ (mg/g) indicates the maximum adsorption capacity of strain FH1; *K*_*F*_ (mg/g) is the Freundlich constant related to adsorption capacity; *K*_*L*_ (L/mg) corresponds to the Langmuir equilibrium constant; n is the Freundlich exponent reflecting adsorption intensity.

### Characterization before and after adsorption

Samples collected before and after adsorption were frozen overnight at −80 °C and lyophilized using a vacuum freeze dryer. Scanning electron microscopy (SEM, MIRA3 LMH, TESCAN, Czech Republic) to observe the surface morphological changes of bacterial cells. X-ray photoelectron spectroscopy (XPS, NEXSA, Thermo Fisher Scientific, USA) to analyze surface chemical states and elemental distribution. Fourier Transform Infrared Spectroscopy (FTIR, IRAffinity-1, Shimazu, Japan) to identify functional group interactions (scanned across 4000-400 cm^−1^).

### Transcriptome sequencing and data analysis

*A. niger* FH1 spores (200 μL) were spread on YPD agar plates supplemented with/without 600 mg/L Ce(III) and overlaid with cellophane membranes. After 3-day incubation (30 °C), mycelia were harvested from membranes, flash-frozen in liquid nitrogen, and stored at −80 °C. Total RNA was extracted using the B518629 Fungal RNA Kit (Sangon Biotech, Shanghai, China) and assessed via 1% agarose gel electrophoresis and spectrophotometry (NanoDrop 2000, Thermo Fisher Scientific, Waltham, MA, USA). Enriched mRNA (Oligo(dT) beads) was fragmented via divalent cation-mediated cleavage (94 °C, 5 min) and reverse-transcribed into cDNA (random hexamers). Libraries were prepared via end repair, adapter ligation (Illumina TruSeq Stranded mRNA) (Illumina, San Diego, CA, USA), size selection (150–300 bp), and PCR amplification (12 cycles). Sequencing (2 × 150 bp paired-end) was performed on an Illumina NovaSeq 6000.

Raw reads were quality-filtered using Trimmomatic (v0.36) and assessed via FastQC (v0.11.2) (Bolger et al., 2014; de Sena Brandine and Smith, 2019). HISAT2 (v2.1.0) aligned reads to the *A. niger* CBS 513.88 reference genome (Kim et al., 2019). Transcript quantification (TPM) and differential expression analysis (DESeq2, v1.12.4) were performed in R. Gene Ontology(GO) and Kyoto Encyclopedia of Genes and Genomes(KEGG) enrichment used topGO (v2.24.0) and clusterProfiler (v3.0.5) (Kanehisa et al., 2020; Klekota et al., 2006; The Gene Ontology Consortium, 2020; Yu et al., 2012). For validation, 10 differentially expressed genes (5 up-/5 downregulated under Ce(III)) were selected. Primers (Supplementary Table 12) were designed via SnapGene (v6.1.1), with GAPDH (An07g01150) as the endogenous control. RT-qPCR reactions (20 μL: 0.4 μL primers, 2 μL cDNA, 10 μL SYBR mix) were run as: 95 °C (3 min); 45 cycles of 95 °C (5 s)/60 °C (30 s).

### Whole-genome bisulfite sequencing (WGBS) and analysis

Genomic DNA was fragmented to ∼250 bp using a Covaris S220 ultrasonicator (Woburn, MA, USA) under standard BS-seq conditions (175W peak power, 10% duty factor, 200 cycles/burst, 55 s) (Williamson et al., 2014). After end-repair and methylated adapter ligation, bisulfite conversion was performed with the EZ DNA Methylation-Gold Kit (Zymo Research, Irvine, CA, USA) per manufacturer protocol. Post-conversion DNA integrity was verified by 2% agarose gel electrophoresis (> 80% fragments within 200–300 bp). Libraries were size-selected (agarose gel), PCR-amplified, and sequenced on an Illumina NovaSeq 6000 platform (150 bp paired-end reads). Raw data were trimmed using TrimGalore (v0.4.4) and quality-checked with FastQC (v0.11.2). BSMAP (v2.9.0) was used to align reads to the reference genome (Xi and Li, 2009). Differentially methylated regions (DMRs) were identified using a 100-bp sliding window with ≥10 × coverage and a methylation difference of ≥25%, consistent with standard practices.

### Statistical analysis

All experiments included three biological replicates. Data are presented as mean ± SD. Model fitting used nonlinear regression (OriginPro 2022, R^2^ > 0.95). The raw data of ITS sequencing of *Aspergillus niger* FH1 screened in this experiment, as well as RNA sequencing and WGBS sequencing under Ce(III) stress, are available in the NCBI BioProject database under accession number PRJNA1331455^2^.

## Results and discussion

### Isolation and identification of the Ce(III)-tolerant fungal strain

**FIGURE 1:**
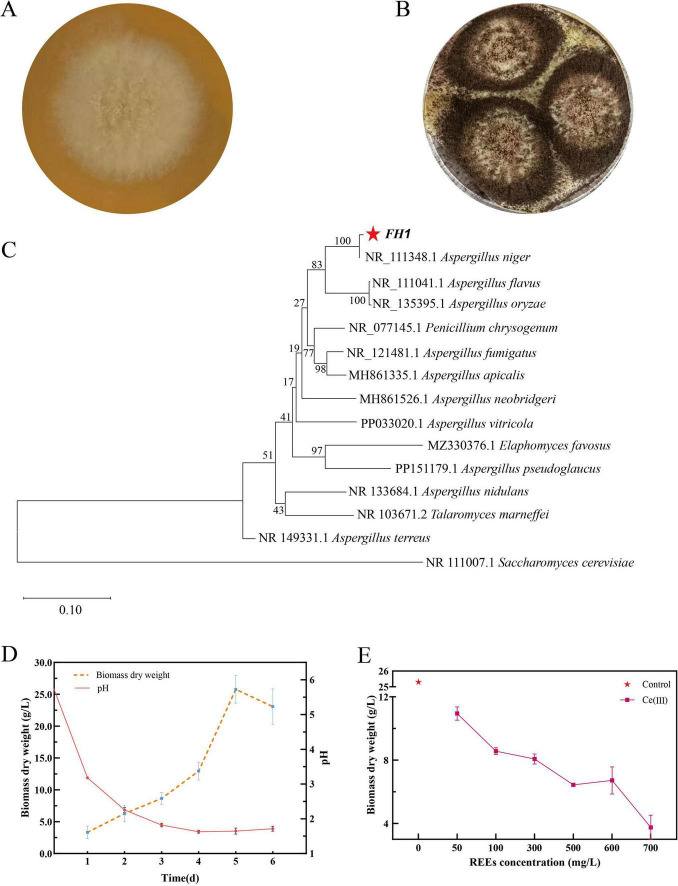
Morphological identification, phylogenetic analysis, growth kinetics, and Ce(III) tolerance of *A. niger* FH1. **(A)** Colony morphology of *A. niger* FH1. **(B)** Spore-forming colony morphology of *A. niger* FH1. **(C)** Phylogenetic tree of *A. niger* FH1. **(D)** Growth curve and corresponding medium pH changes. **(E)** Dose-dependent growth inhibition by Ce(III) (50-700 mg/L). The symbol * in panel **(C)** represents *Aspergillus niger* strain FH 1, and in panel **(D)** represents the dry biomass weight of FH1 at a Ce(III) concentration of 0 mg/L.

To isolate rare earth-tolerant strains, soil samples from a rare-earth mining area in China were screened using media supplemented with 50 mg/L Ce(III). A dominant fungal strain (designated FH1) was purified on PDA plates. Morphological analysis revealed that FH1 colonies transitioned from white (24 h post-inoculation) (Figure 1A) to black spore-forming structures with radial cracks and fringed edges at maturity (Figure 1B). Phylogenetic analysis of the ITS region confirmed FH1 as *Aspergillus niger* (100% bootstrap support, Figure 1C). Growth kinetics of FH1 in YPD medium showed a lag phase (Days 1–3), followed by exponential growth peaking at 27.45 g/L biomass on Day 5. The pH of the culture medium gradually decreases with cell growth, showing an overall negative correlation with biomass (Figure 1D). Exposure to Ce(III) (50–700 mg/L) significantly inhibited growth in a dose-dependent manner (Figure 1E). At 700 mg/L Ce(III), biomass decreased by 85% compared to the control, establishing 600 mg/L as the maximum tolerated concentration.

### Optimization of Ce(III) adsorption by *A. niger* FH1 and its adsorption performance and kinetics

#### pH dependence

**FIGURE 2:**
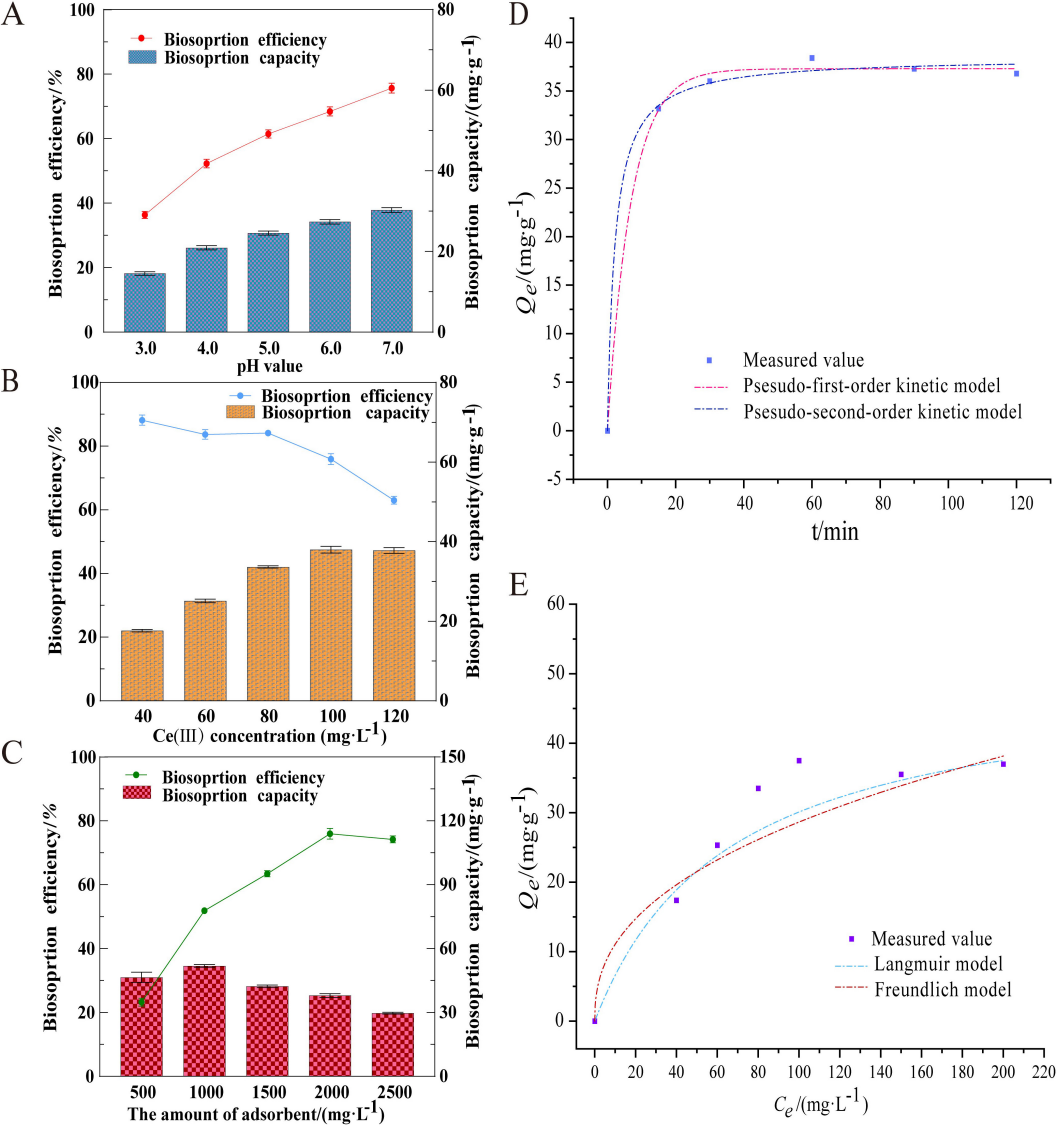
Optimization of biosorption parameters and mechanistic modeling for Ce(III) removal by *A. niger* FH1: **(A–C)** Effects of pH, initial Ce(III) concentration, and biomass dosage on adsorption performance; **(D)** kinetic modeling of the adsorption process; **(E)** adsorption isotherms fitted with Langmuir and Freundlich models.

pH critically influences adsorption efficiency by modulating surface functional group activity, metal ion speciation, and competitive binding (Buayam et al., 2019). As shown in Figure 2A, both adsorption rate and equilibrium capacity of *A. niger* FH1 increased with pH (2.0–7.0). At low pH (2.0–4.0), excess H_3_O^+^ ions protonated carboxyl (-COOH), phosphate (-PO3-4), and amine (-NH_2_) groups, creating electrostatic repulsion against cationic Ce(III). Above pH 5.0, deprotonation of these groups enhanced Ce(III) binding via electrostatic attraction and ligand exchange (Elkomy and Rizk, 2019). However, at pH >7.0, Ce(III) precipitated as Ce(OH)_3_, reducing soluble ion availability. Thus, pH 7.0 was identified as optimal.

#### Initial ion concentration

Adsorption capacity increased with Ce(III) concentration (20–100 mg/L), peaking at 42.61 mg/g (Figure 2B). With the increase in the initial concentration of rare earth ions, the collision probability between rare earth ions and the effective adsorption sites on the bacterial strain also increases. However, the number of adsorption sites available on the bacterial surface for rare earth ions is limited. As a result, the adsorption of rare earth ions by the strain gradually reaches saturation. This may explain why the adsorption capacity at equilibrium increases with the initial concentration of rare earth ions and why there is a maximum value (Gupta et al., 2006). The plateau suggests finite binding sites on fungal biomass, with maximal utilization achieved at 100 mg/L.

#### Biomass dosage

The biomass dosage is a critical determinant of the number of metal-binding sites in the adsorption system and significantly influences adsorption efficiency (Jamir et al., 2024). As shown in Figure 2C, the adsorption efficiency of *A. niger* FH1 increased proportionally with biomass dosage, reaching a maximum of 76.84% at 2000 mg/L. However, the equilibrium adsorption capacity (*q*_*e*_) inversely correlated with biomass dosage. Elevated biomass concentrations induced shielding effects, where peripheral hyphae obstructed access to internal binding sites, thereby reducing effective site availability and lowering adsorption efficiency (Gupta et al., 2019). Furthermore, at low biomass dosages, limited binding sites prompted competitive ion occupancy, resulting in complete site saturation. Although lower biomass concentrations exhibited higher adsorption capacity per unit biomass, increased biomass diluted site utilization efficiency, leading to reduced overall adsorption capacity (Das and Das, 2013). Based on these findings, the optimal biomass dosage for *A. niger* FH1 was determined to be 2000 mg/L.

The biosorption process of *A. niger* FH1 was fitted to pseudo-first-order and pseudo-second-order kinetic models, with results and parameters shown in Figure 2D and Supplementary Table 1. The pseudo-first-order model assumes adsorption rate is governed solely by physical diffusion, while the pseudo-second-order model posits chemical mechanisms involving electron sharing or transfer between adsorbent and adsorbate (Ezzati et al., 2024; Ho, 2006). As shown in Figure 2D, the adsorption process exhibited biphasic kinetics: rapid uptake within 15 min (89.12% of maximum capacity), followed by gradual equilibration reaching saturation at 60 min. Both models yielded high correlation coefficients (*R*^2^> 0.99), with predicted equilibrium capacities (*q*_*e*_) of 37.29 mg/g (pseudo-first-order) and 38.46 mg/g (pseudo-second-order), closely matching experimental values. This suggests a hybrid mechanism combining physical adsorption (diffusion-limited) and chemisorption (surface reaction).

Further analysis using Langmuir and Freundlich isotherm models (Figure 2E and Supplementary Table 2) revealed superior fit for the Langmuir model (*R*^2^ = 0.9705) over Freundlich (*R*^2^ = 0.9579), indicating monolayer adsorption on homogeneous surfaces. The Freundlich model’s lower performance implies limited contribution from heterogeneous multilayer adsorption (Basu et al., 2022). This aligns with prior studies showing *A. niger* adsorption of Y(III) and Nd(III) also conforms to the Langmuir model (Wierzba, 2017), reinforcing the prevalence of monolayer binding in fungal biosorption systems.

While typical Ce(III) concentrations in environmental and industrial effluents are generally lower than the 600 mg/L tolerance threshold reported for *A. niger* FH1, this high resilience enhances its applicability in scenarios involving concentrated REE streams. In acid mine drainage (AMD), a common source of REE contamination from mining activities, Ce(III) levels typically range from 0.05 to 0.3 mg/L in neutral mine drainage to higher values such as 3.28 mg/L in specific acidic samples with total REE concentrations occasionally reaching up to 25.5 mg/L (Hermassi et al., 2022; Shahhosseini et al., 2017). These concentrations are influenced by factors like pH, geology, and seasonal evaporation, often showing enrichment in light REEs like Ce (Obregón-Castro et al., 2023). For e-waste leachates, another key REE source amid growing electronic recycling demands, Ce(III) can reach approximately 29 mg/L in processed industrial slags, though ambient landfill leachates are usually lower (Vapnik et al., 2025). However, in controlled hydrometallurgical leaching processes for REE recovery from e-waste—such as acid-based extraction from magnets or circuit boards—concentrations can be deliberately elevated to hundreds of mg/L to optimize yield (Liang et al., 2024; Peelman et al., 2016). The exceptional tolerance of *A. niger* FH1 thus positions it as a robust biosorbent for not only dilute effluents but also intensified recovery systems, where pre-concentration steps or variable high-load inputs could exceed typical ambient levels, reducing risks of microbial inhibition and enabling scalable, sustainable REE remediation.

Biosorption capacities for REE vary widely across microbial and biomass-based adsorbents, often influenced by factors such as pH, initial ion concentration, and biosorbent type. For instance, Bacillus licheniformis has been reported to achieve a Ce(III) adsorption capacity of 38.93 mg/g with 97% removal efficiency, fitting the Freundlich isotherm and pseudo-second-order kinetics, under conditions where phosphate and carboxyl groups facilitate binding (Cheng et al., 2016). This is slightly lower than the 42.61 mg/g observed here, though the higher removal percentage may stem from differences in biosorbent dosage or initial concentrations. Similarly, engineered *Caulobacter crescentus* displaying lanthanide-binding tags showed a Ce(III) capacity of approximately 8.58 mg/g (without competing Ca^2 +^ ions) at pH 5–6, with efficiencies up to 93% for related REEs like Tb(III), but capacities dropped in the presence of divalent cations (Park et al., 2016). Earlier studies on *A. niger* for Ce(III) reported capacities as low as 1.74 mg/g at initial concentrations of 100 mg/L, with increases linked to metal ion availability (Rezk and Morse, 2023). In multi-REE systems, *A. niger* strains reached ∼75% efficiency for La^3 +^, Sm^3 +^, Y^3 +^, Nd^3 +^, and Er^3 +^, but specific capacities remained unreported (Zhou et al., 2024). Broader fungal applications, such as *Rhizopus arrhizus* for Cu(II), yielded 97.32% efficiency and capacities up to 97.32 mg/g at 80 mg/L, while white-rot fungi like *Phanerochaete chrysosporium* achieved 110 mg/g for Pb(II) (Chauhan et al., 2020; Yetis et al., 1998).

Although exopolysaccharides (EPS) and extracellular polymeric substances were not analyzed in this study, they likely contribute to *A. niger*FH1’s Ce(III) biosorption. Fungal EPS matrices contain carboxyl, hydroxyl, and phosphate groups that facilitate metal cation complexation via ion exchange. In *Aspergillus* species, EPS production under metal stress enhances adsorption—achieving 88% removal for Ag nanoparticles with pseudo-second-order kinetics (*R*^2^ = 0.98) consistent with our findings (Gomaa et al., 2022). EPS-mediated surface complexation also drives Cr adsorption in *A. niger* (Xu et al., 2022). While REE-specific fungal EPS data are limited, analogous cyanobacterial studies attribute 16–41% of light REE adsorption (e.g., Ce(III)/Nd(III)) to EPS polysaccharide chelation (104–138 mg/g capacity) (Paper et al., 2023). FH1’s high Ce(III) tolerance (600 mg/L) may thus derive from EPS-supported extracellular binding. Future EPS characterization via FTIR/EDS would clarify these mechanisms for biosorbent optimization.

### Characterization analysis and mechanism study of Ce(III) adsorption by *A. niger* FH1

#### SEM analysis

**FIGURE 3:**
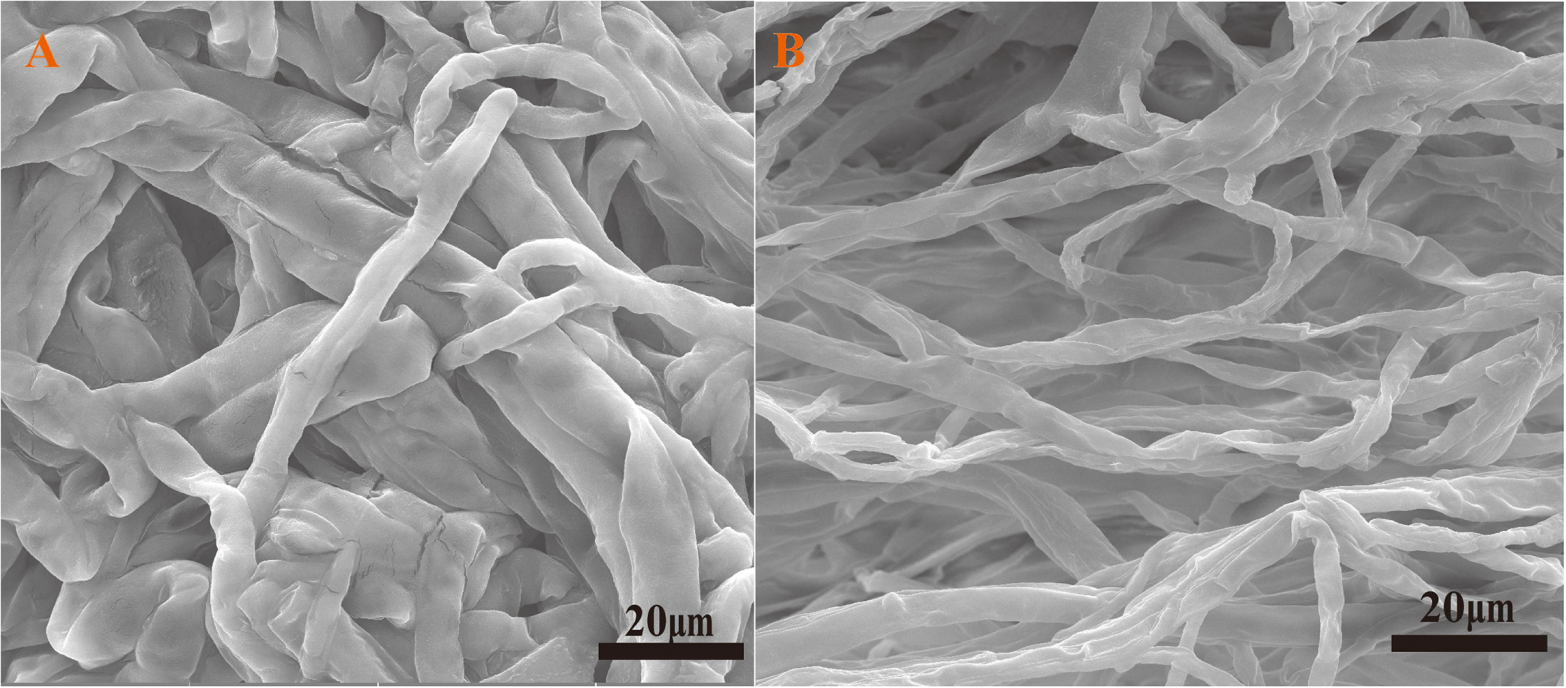
SEM images of *A.niger* before **(A)** and after **(B)** biosorption.

Scanning Electron Microscopy (SEM) was employed to observe morphological alterations in *A. niger* FH1 under Ce(III) stress. As shown in Figure 3A, untreated *A. niger* FH1 cells exhibited a relatively smooth surface with irregular grooves. In contrast, Ce(III)-exposed cells displayed a roughened surface, cellular invaginations, reduced intercellular spacing, and pronounced aggregation (Figure 3B). These morphological alterations likely correlate with cellular responses to rare earth ion stress. Previous studies suggest that microbes mitigate metal toxicity by modifying surface topography (e.g., inducing wrinkles or folds) to reduce effective surface area (Priyanka and Dwivedi, 2023), a strategy consistent with the observed FH1 adaptations.

#### FTIR analysis

**FIGURE 4:**
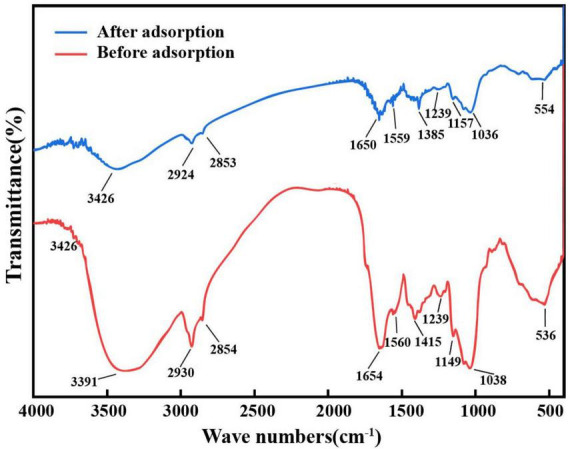
FT-IR spectra of *A.niger* before and after Ce(III) biosorption.

Fourier transform infrared spectroscopy (FTIR) spectra of *A. niger* FH1 before and after Ce(III) adsorption are shown in Figure 4. Prior to adsorption, the broad peak at 3391 cm^−1^ corresponds to overlapping O-H (hydroxyl) and N-H (amine) stretching vibrations from polysaccharides and amino acids (Hisada and Kawase, 2018; Oliveira et al., 2014). Post-adsorption, this peak shifted to 3426 cm^−1^. The C-H stretching vibrations (2800–3000 cm^−1^) exhibited a shift from 2930 cm^−1^ to 2924 cm^−1^ (Guiza, 2017). Peaks at 1654 cm^−1^ (C = O stretching of carboxyl/amide groups) (Wang F. et al., 2017) and 1415 cm^−1^ (C-O symmetric stretching of -COO^–^) (Roozegar and Behnam, 2018) also shifted, while phosphate-related peaks (1038 cm^−1^ and 1149 cm^−1^) (Blackwell et al., 1995) showed reduced intensity. These spectral changes confirm the involvement of amine (-NH_2_), hydroxyl (-OH), carboxyl (-COOH), and phosphate (-${\text{PO}}_{4}^{3-}$) groups in Ce(III) binding. Studies indicate that these functional groups provide adsorption sites for metal ions through ligand exchange and coordination reactions (Arslan and Kütük, 2023), suggesting a similar mechanism for Ce(III) adsorption by *A. niger* FH1.

#### XPS analysis

**FIGURE 5:**
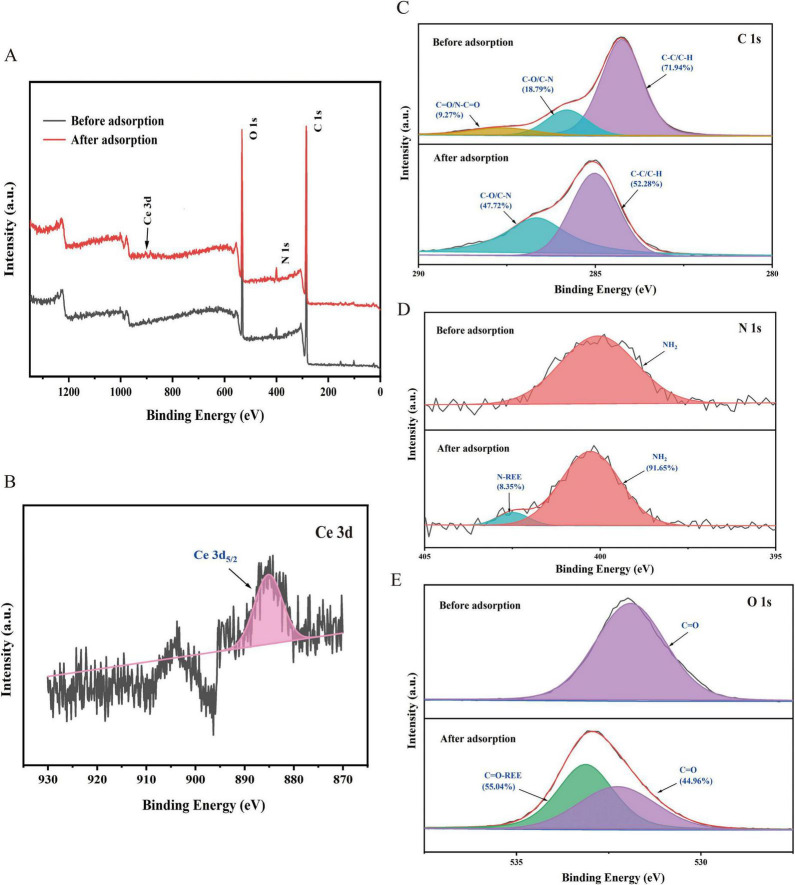
XPS analysis of *A.niger*: survey spectra **(A)**, Ce 3d spectra **(B)**, C 1 s spectra before and after Ce(III) biosorption **(C)**, N 1 s spectra before and after Ce(III) biosorption **(D)**, and O 1 s spectra before and after Ce(III) biosorption **(E)**.

In order to further validate the FTIR characterization results, XPS was employed to analyze the elemental composition and chemical valence states of *A. niger* FH1 before and after adsorption. In the XPS survey spectrum of *A. niger* FH1 after adsorption (Figure 5A), the characteristic peak of Ce 3d was observed, confirming that Ce(III) can be adsorbed by *A. niger* FH1. As illustrated in Figure 5B, the Ce 3d spectrum of *A. niger* FH1 following adsorption exhibited a single peak corresponding to Ce 3d5/2 (884.42 eV), indicative of the trivalent state of Ce. In the C 1s spectra, the peaks before adsorption consist of three components: C-C/C-H, C-O/C-N, and C = O/N-C = O (Bertagnolli et al., 2014; Cid et al., 2018). Subsequent to the adsorption process, a decline in the peak areas of C-C/C-H and C = O/N-C = O in the *A. niger* FH1 group was observed, accompanied by an increase in the C-O/C-N peak area. This finding suggests that C-C/C-H, C-O/C-N, and C = O/N-C = O participated in the adsorption process of *A. niger* FH1 (Figure 5C). As demonstrated in Figure 5D, prior to the adsorption process, only the characteristic peak of -NH2 (399.85 eV) (Hisada and Kawase, 2018) was observed in *A. niger* FH1. Subsequent to the adsorption process, a novel characteristic peak emerged at 402.47 eV. This peak has been documented to be linked with the complexation reaction between nitrogen atoms and rare earth ions (Gougousi and Chen, 2008). The decrease in the peak area of the -NH2 group indicates that during adsorption, amine groups form stable complexes with La(III) and Ce(III) to participate in the process. In the O 1s spectrum of the *A. niger* FH1 group (Figure 5E), only one peak at 531.04 eV, corresponding to C = O, was observed prior to adsorption (Cao et al., 2021). Subsequent to the adsorption process, a novel characteristic peak emerged at 533.13 eV, which was ascribed to the coordination bond formed between C = O and rare earth ions (Ramrakhiani et al., 2017).

The combined FTIR and XPS results demonstrate that Ce(III) adsorption by *A. niger* FH1 involves diverse functional groups (-NH_2_, -OH, -COOH, -${\text{PO}}_{4}^{3-}$) through ligand exchange and coordination. Surface amine groups form stable Ce-N complexes, while carboxyl and hydroxyl groups participate in Ce-O bonding. Phosphate groups, though less prominent, contribute to electrostatic interactions. This multi-modal binding strategy aligns with fungal metal resistance mechanisms, where surface functionalization mitigates metal toxicity while enhancing adsorption capacity.

### Transcriptomic response of *A. niger* FH1 to Ce(III) stress

**FIGURE 6:**
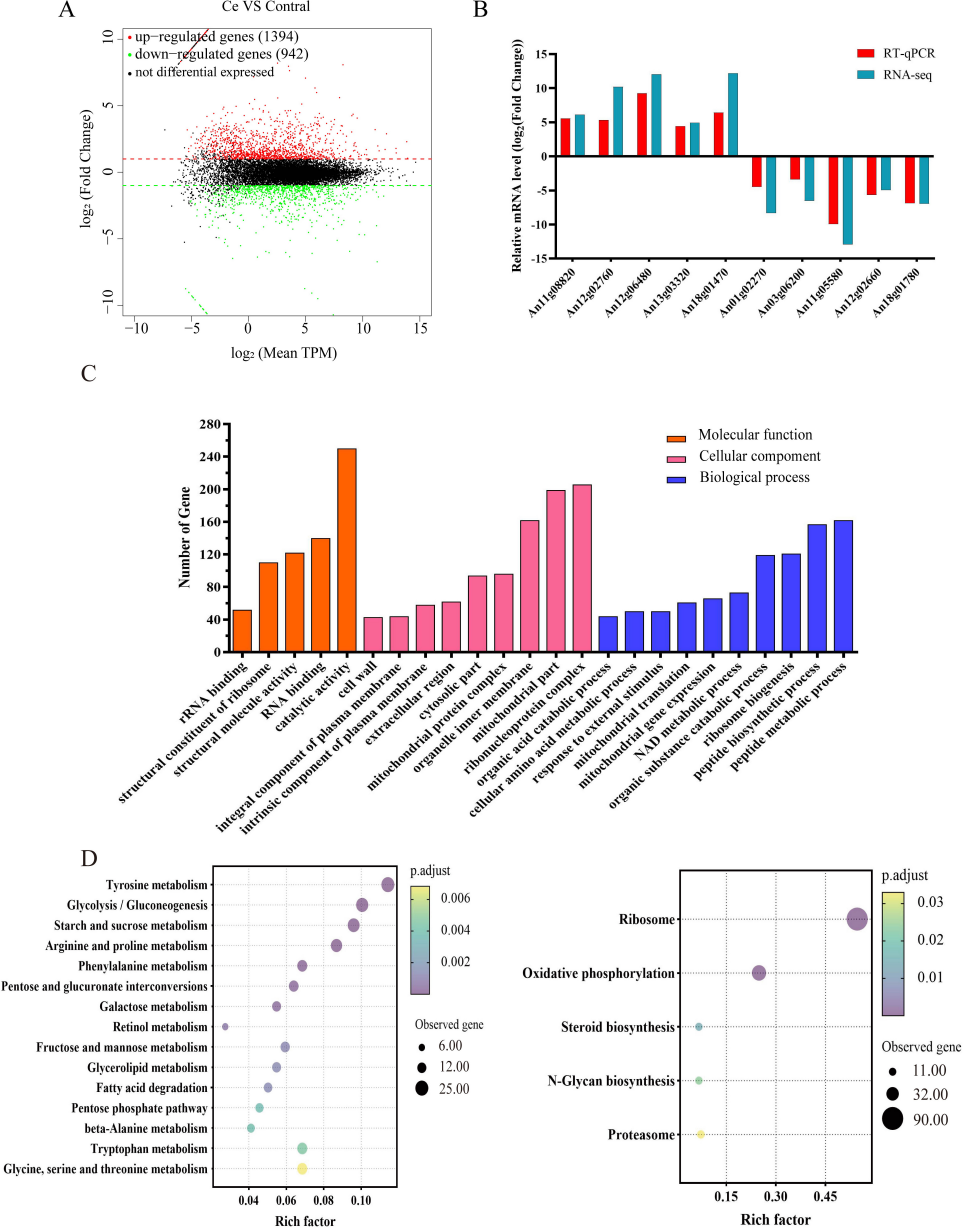
Transcriptomic response of *A. niger* FH1 to Ce(III) stress. **(A)** Volcano plot of DEGs under Ce(III) stress. **(B)** RT-qPCR validation of DEGs under Ce(III) stress. *GAPDH* (*An07g01150*) was used as the reference gene. **(C)** GO enrichment analysis of DEGs under Ce(III) stress. **(D)** KEGG pathway enrichment analysis of upregulated (left) and downregulated (right) genes under Ce(III) stress.

RNA sequencing was performed on *A. niger* FH1 exposed to 600 mg/L Ce(III) (treatment group) or 0 mg/L Ce(III) (control), with three biological replicates per condition. Libraries were prepared from mid-log phase cultures, yielding 48.73–72.15 million raw reads per sample. After stringent quality filtering (Q30 > 93%), clean reads (7.47–10.10 Gb) exhibited >89% alignment to the *A. niger* CBS 513.88 reference genome, confirming data robustness (Supplementary Table 3; Pel et al., 2007). Using thresholds of | log2(fold change)| ≥ 1 and Benjamini-Hochberg adjusted *p* < 0.01, we identified 3,733 differentially expressed genes (DEGs): 2,459 upregulated and 1,274 downregulated (Figure 6A). RT-qPCR validation of 10 randomly selected DEGs (5 upregulated, 5 downregulated) confirmed strong concordance with RNA-seq trends (Figure 6B).

Gene Ontology (GO) enrichment analysis (Benjamini-Hochberg adjusted *p* < 0.01) identified 24 significantly enriched terms for *A. niger* FH1 differentially expressed genes (DEGs) under 600 mg/L Ce(III) stress, with prominent associations in “cellular amino acid metabolism” (GO:0006520), “organic acid catabolism” (GO:0016054), and “mitochondrial gene expression” (GO:0140053) (Figure 6C). Complementary KEGG pathway analysis revealed metabolic shifts: upregulated DEGs were enriched in glycolysis (ko00010), phenylalanine metabolism (ko00360), tyrosine metabolism (ko00350), and arginine/proline metabolism (ko00330) (Figure 6D) (Left), suggesting enhanced activity in stress-responsive metabolic pathways. Conversely, downregulated DEGs were associated with oxidative phosphorylation (ko00190) and ribosome biogenesis (ko03010) (Figure 6D) (Right), indicating a potential reduction in energy-intensive processes under Ce(III) exposure. These enrichment patterns suggest that Ce(III) stress may influence amino acid metabolism and mitochondrial-related gene expression, potentially as part of a broader adaptive response, though direct impacts on cellular homeostasis or function, such as growth defects, were not assessed in this study. Further discussion of these pathways is provided below.

### Response mechanism of *A. niger* to Ce(III) stress

#### Oxidative phosphorylation

Oxidative phosphorylation primarily occurs in the mitochondria of fungi and is also known as the mitochondrial respiratory chain. It serves as a crucial pathway for ATP synthesis and energy production in eukaryotes (Wang et al., 2021). The oxidative phosphorylation system consists of five key enzymes: Nicotinamide Adenine Dinucleotide (NADH) dehydrogenase, ATP synthase (ATPase), Succinate dehydrogenase (SDH), Cytochrome c oxidase, and the Cytochrome bc1 complex. These enzymes work in coordination to couple electron transport across the inner mitochondrial membrane with ATP production (Rutter and Hughes, 2015). Studies have shown that high concentrations of metal ion stress can elevate intracellular levels of reactive oxygen species (ROS) in eukaryotic cells, leading to damage in the mitochondrial respiratory chain and uncoupling of oxidative phosphorylation (Mansoor et al., 2023). In this experiment, the KEGG enrichment results indicated that the oxidative phosphorylation pathway was downregulated under Ce(III) stress. The related differentially expressed genes (DEGs) are listed in Supplementary Table 4, including genes encoding ATP synthase (*An01g04630*, *An01g04930*, *An12g04950*, *An16g08550*, *An01g10880*, *An07g06560*, and *An14g00820*), cytochrome c (*An02g01830*), cytochrome c oxidase (*An14g04170*, *An02g09930*, *An07g07390*, *An11g10200*, *An02g01720*, *An09g03990*, *An04g01560*, *An02g04330*, *An11g02430*, and *An02g12620*), cytochrome c reductase (*An14g04080*, A*n01g06180*, *An09g06650*, *An04g05220*, *An04g01200*, and *An08g06550*), and NADH dehydrogenase (*An11g06200*, *An04g00060*, *An18g05670*, *An04g05640*, and *An12g04780*), all of which were downregulated.

In mitochondria, NADH dehydrogenase, cytochrome c reductase, and cytochrome c oxidase are core components of the electron transport chain in mitochondrial respiration. Through coordinated action, they facilitate electron transfer via the following pathway: First, NADH dehydrogenase catalyzes the oxidation of NADH, transferring two high-energy electrons to coenzyme Q and generating NAD^+^ and reduced coenzyme Q. Next, cytochrome c reductase transfers electrons from reduced coenzyme Q to cytochrome c stepwise through the Q-cycle mechanism. Finally, cytochrome c oxidase delivers electrons from cytochrome c to oxygen (O_2_). During electron transfer, these three enzymes simultaneously pump protons (H^+^) into the mitochondrial intermembrane space to establish a proton gradient, thereby providing the driving force for ATP synthase (Lavín et al., 2008). Several studies have shown that under high concentrations of Cd(II), Cu(II), and Pb(II) stress, the activities of NADH dehydrogenase, cytochrome c reductase, and cytochrome c oxidase in eukaryotic cells decrease, leading to disruptions in energy metabolism (Castro-Guerrero et al., 2008; Doğanlar et al., 2014). Additionally, research has demonstrated that overexpression of genes encoding cytochrome c oxidase can enhance the oxidative stress tolerance of Saccharomyces cerevisiae cells (Keerthiraju et al., 2019). ATP synthase utilizes the proton gradient across the inner mitochondrial membrane to catalyze the synthesis of ATP from ADP and inorganic phosphate (Pi). This is the final step of oxidative phosphorylation and the primary pathway for ATP production in cells. Studies have shown that fungal ATP synthase activity is affected under metal ion stress. For example, in Penicillium exposed to high concentrations of Cu(II), the expression levels of genes encoding ATP synthase were also observed to decrease, which is considered a key factor in Cu(II)-induced impairment of ATP production efficiency (Xu et al., 2015).

Based on the above, this study hypothesizes that the toxicity mechanism of Ce(III) toward *A. niger* involves: inhibiting the expression levels of genes encoding key enzymes in oxidative phosphorylation, leading to decreased activity of these enzymes, thereby reducing ATP synthesis efficiency and ultimately causing disruption of cellular energy metabolism.

#### Glycolysis

The glycolysis (or EMP) is a process in which glucose is broken down into pyruvate, accompanied by the generation of ATP and NADH. It is closely related to energy supply, carbon metabolism regulation, and environmental adaptation, serving as a core metabolic process in fungi (Jia et al., 2024). In our study, the glycolytic pathway was upregulated under Ce(III) stress, with related genes listed in Supplementary Table 5. Among them, genes encoding glyceraldehyde-3-phosphate dehydrogenase (GAPDH) (*An16g01830*), hexokinase (HK) (*An02g14380*), and enolase (ENO) (*An18g06250*) were upregulated. The glycolytic pathway consists of ten reactions, with HK being the key enzyme catalyzing the conversion of glucose to glucose-6-phosphate—the first glycolytic reaction—thus playing a crucial role in regulating the initiation rate of glycolysis (Fuentes-Lemus et al., 2025). GAPDH participates in the sixth reaction of glycolysis, catalyzing the conversion of glyceraldehyde-3-phosphate to 1,3-disphosphoglycerate, generating NADH, which provides electrons for the mitochondrial respiratory chain (Choe et al., 2025). Enolase drives the subsequent ATP-generating reaction catalyzed by pyruvate kinase (PK) by catalyzing the conversion of 2-phosphoglycerate to the high-energy phosphocompound phosphoenolpyruvate in the ninth step (Kierans and Taylor, 2024). In eukaryotes, the activity of these three enzymes is affected under metal ion stress. For example, HK activity in Chlorophyta has been shown to increase under Cu(II) treatment (Laporte et al., 2020); GAPDH accumulates significantly in Arabidopsis under Cd(II) stress (Vescovi et al., 2013); and the gene encoding enolase is upregulated in Phytolacca americana exposed to Cd(II), making it a candidate Cd-resistance gene (Zhao et al., 2023). The upregulation of key glycolytic enzyme genes indicates that the glycolytic pathway in *A. niger* is accelerated under Ce(III) stress, providing the energy necessary to drive other detoxification processes. This accelerated glycolysis partially compensates for the deleterious effects of the observed oxidative phosphorylation inhibition under Ce(III) stress.

#### Pentose phosphate pathway (PPP)

Pentose phosphate pathway not only serves as a complementary pathway for energy metabolism but also plays a crucial role in biosynthesis, antioxidation, and environmental adaptation by providing NADPH (reduced form of nicotinamide-adenine dinucleotide phosphate) and ribose-5-phosphate. Under Ce(III) stress, the PPP is upregulated, with relevant genes listed in Supplementary Table 6, including the gene *An11g06120* encoding 6-phosphogluconate dehydrogenase (6PGDH) and the gene *An08g06570* encoding transketolase (TK), both of which are upregulated. The PPP consists of oxidative and non-oxidative phases: the oxidative phase primarily generates NADPH, while the non-oxidative phase participates in the synthesis of pentoses and other sugars (Ma et al., 2015). 6PGDH is a key enzyme in the oxidative phase of PPP, catalyzing the decarboxylation of 6-phosphogluconate (6PG) to produce ribulose-5-phosphate (Ru5P), a reaction that also generates NADPH (Reyes et al., 2024). Studies have shown that 6PGDH activity increases under oxidative stress (e.g., elevated intracellular ROS levels), enhancing NADPH supply in cells. NADPH, in turn, protects cells from oxidative damage by maintaining the reduced state of the critical antioxidant glutathione (GSH) (Tian et al., 2021). TK catalyzes carbon chain transfer between pentoses and tetroses, facilitating the production of fructose-6-phosphate (F6P) and glyceraldehyde-3-phosphate (G3P). This process links PPP with EMP pathway, improving carbon metabolic flexibility (Clasquin et al., 2011). According to Sezer et al., exposure to high concentrations of Cu(II) increases TK activity in *Phanerochaete chrysosporium*, which is considered a response to Cu(II)-induced oxidative stress (Okay et al., 2020).

Based on these findings, it is hypothesized that upregulating the expression of genes encoding 6PGDH and TK to enhance their activity represents a response mechanism of *A. niger* to Ce(III)-induced oxidative stress.

#### Amino acid metabolism

According to the KEGG enrichment results, under Ce(III) stress, multiple amino acid metabolism-related pathways were upregulated, including phenylalanine metabolism, arginine and proline metabolism, glycine, serine and threonine metabolism, and tyrosine metabolism (Supplementary Tables 7–10).

Phenylalanine metabolism is not only a part of fundamental energy metabolism but also a key pathway for fungal adaptation to environmental stress. Under Ce(III) stress, the gene *An08g07740* encoding phenylalanine ammonia-lyase (PAL) is upregulated. Phenylalanine, catalyzed by PAL, undergoes deamination to form cinnamic acid, a crucial precursor for phenolic compound synthesis in eukaryotes. These phenolic compounds act as non-enzymatic antioxidants to scavenge ROS induced by abiotic stress (Hyun et al., 2011). Increased PAL activity has been observed in wheat (*Triticum aestivum*) under high-concentration Cu(II) stress and in grape leaves (*Vitis quinquangularis*) under Al(III) stress (Jańczak-Pieniążek et al., 2022; Wang Q. et al., 2021). By enhancing the expression of genes related to PAL encoding to increase the production of non-enzymatic antioxidants, *A. niger* can indirectly maintain the balance between intracellular oxidants and antioxidants under Ce(III)-induced oxidative stress.

Under Ce(III) stress, the genes *An14g01190* and *An02g07250* encoding arginase (ARG) and the gene *An01g01520* encoding pyrroline-5-carboxylate reductase (P5CR) were all upregulated. ARG catalyzes the conversion of arginine to ornithine, serving as a key enzyme in proline synthesis via the ornithine pathway, while P5CR plays a crucial role in proline synthesis through the glutamate pathway (Liang et al., 2014). Proline accumulation is one of the mechanisms by which various eukaryotes resist heavy metal toxicity (Mohammadi Alagoz et al., 2023). Increased proline production under metal stress such as Pb and Cu has been reported in multiple eukaryotic species (Dachuan and Jinyu, 2021; Siddiqi and Husen, 2011). Furthermore, several proline-mediated heavy metal stress response mechanisms have been studied. Free proline can act as a compatible osmolyte, functioning as an osmoprotectant and protein stabilizer to maintain protein structure. Additionally, it can reduce ROS accumulation by serving as an inhibitor of lipid peroxidation, a scavenger of hydroxyl radicals, and a singlet oxygen scavenger (Mukherjee et al., 2010; Signorelli et al., 2014). Under Ce(III) stress, the upregulation of genes encoding ARG and P5CR, two key enzymes involved in proline synthesis, promotes proline production and accumulation. Proline then participates in processes such as ROS scavenging and maintaining cellular osmotic balance, which may represent an important detoxification mechanism of *A. niger* against Ce(III).

In the glycine, serine, and threonine metabolic pathways, the gene *An08g03070* encoding aminomethyltransferase (AMT) and the gene *An07g00680* encoding threonine aldolase (TA) were upregulated. AMT is a key enzyme in the glycine cleavage system (GCS), the core enzyme complex responsible for glycine degradation, also known as glycine cleavage system T protein. Its function is to catalyze the conversion of tetrahydrofolic acid (THF) to 5,10-methylenetetrahydrofolate (5,10-CH2-THF) (Timm et al., 2018). Studies have shown that 5,10-CH2-THF generated by GCS participates in the methionine cycle, thereby promoting the synthesis of S-adenosylmethionine (SAM) (Young et al., 2022). SAM, in turn, provides methyl groups for the methylation modification of enzymes involved in the synthesis of glutathione (GSH), an important cellular antioxidant, indirectly enhancing GSH production (Wang et al., 2022). GSH is composed of glycine, glutamate, and cysteine. It has been reported that under metal ion stress, cells increase glycine production to supply sufficient raw materials for GSH synthesis, thereby indirectly eliminating excess ROS induced by oxidative stress (Sharma et al., 2024). TA functions to cleave threonine into glycine and acetaldehyde (Fesko, 2016). The upregulation of its encoding gene under Ce(III) stress may be related to the increased demand for glycine to synthesize GSH when cells are under oxidative stress.

The genes encoding tyrosinase (TYR) (*An09g02980*, *An09g05130*, and *An15g07670*) and 4-hydroxyphenylpyruvate dioxygenase (HPPD) (*An04g01280*) in the tyrosine metabolic pathway were upregulated. TYR is a key enzyme in melanin biosynthesis, catalyzing the oxidation of tyrosine to L-dopa, which further polymerizes to form melanin (Eisenman and Casadevall, 2012). In microbial systems, melanin is known to function as an antioxidant, UV protectant, thermoregulator, and metal chelator (Słominski et al., 1988). Studies have shown that exposure to certain metals such as Fe(III), Zn(II), and Cu(II) can activate the melanin synthesis pathway, and melanin production helps fungi adapt to metal stress (McGraw, 2003). Functional groups such as phenolic hydroxyl, hydroxyl, carboxyl, and amino groups in fungal cell wall melanin provide multiple effective adsorption sites for metal ions (El-Gazzar et al., 2025). According to Perdigão Cota de Almeida et al. (2021), in a secretomics study of *A. niger* under copper stress, melanin production was found to increase under Cu(II) induction, leading to melanization of the *A. niger* cell wall and enhancing its Cu(II) adsorption capacity. HPPD is also involved in melanin biosynthesis (Ruzafa et al., 1994). Additionally, reports indicate that in various eukaryotes, HPPD can negatively regulate intracellular ROS levels by modulating tyrosine catabolism (Liang et al., 2020; Wang W. et al., 2020). In summary, the upregulation of TYR and HPPD genes promotes melanin synthesis, thereby enhancing the antioxidant defense and Ce(III) adsorption capacity of *A. niger*.

**FIGURE 7:**
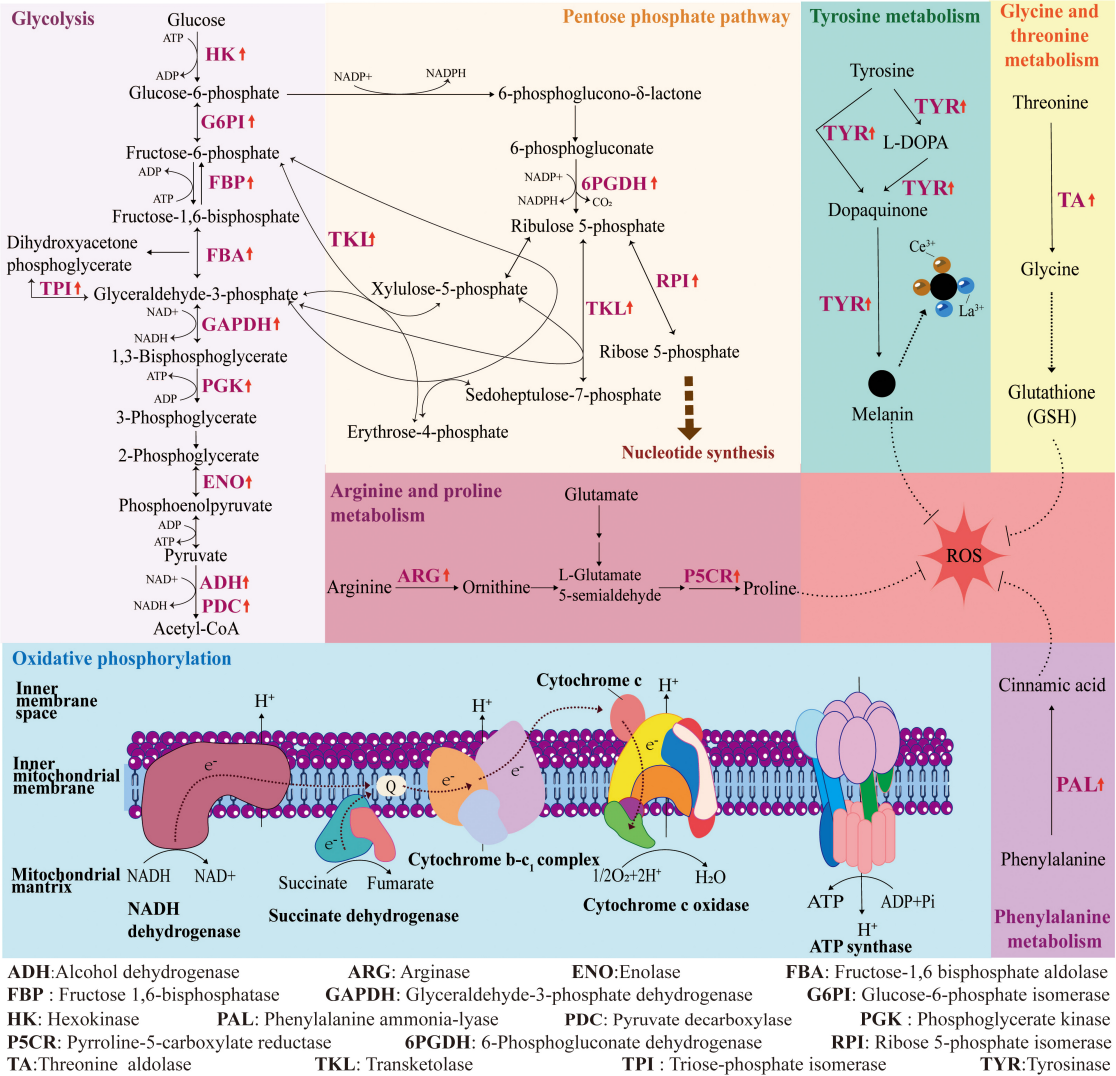
Integrated metabolic reprogramming in *A. niger* FH1 under Ce(III) stress.

In summary (Figure 7), under Ce(III) stress, *A. niger* enhances the synthesis of key antioxidant substances to counteract oxidative stress by regulating the expression of critical genes in phenylalanine metabolism, proline metabolism, as well as glycine and threonine metabolism. The upregulation of genes encoding key enzymes in tyrosine metabolism increases melanin production, thereby improving the cell’s adsorption capacity for rare earth ions. Additionally, the expression of genes encoding key enzymes in oxidative phosphorylation is suppressed, reducing ATP synthesis efficiency and triggering energy metabolism disorders. Conversely, the upregulation of genes encoding multiple key enzymes in glycolysis and the pentose phosphate pathway provides more driving energy and essential raw materials for detoxification-related physiological processes.

### Genome-wide DNA methylation analysis of *A. niger* FH1 under Ce(III) stress

**FIGURE 8:**
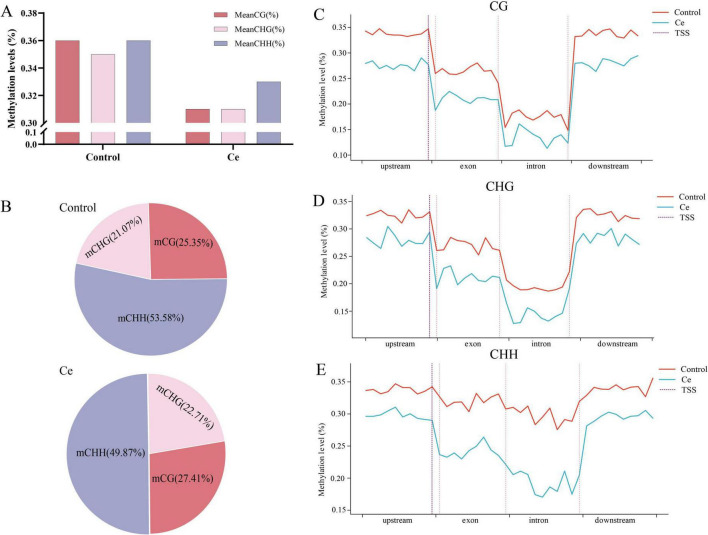
Genome-wide DNA methylation dynamics in *A. niger* FH1 under Ce(III) stress. **(A)** Methylation levels in CG, CHG, and CHH contexts. **(B)** Distribution of cytosine methylation types (CG, CHG, CHH). **(C-E)** TSS-proximal methylation declined sharply in all contexts (CG, CHG, CHH) under stress. Gene-body methylation was lower in CG/CHG (vs. flanking regions), contrasting with uniform CHH in genic/intergenic zones. No significant DMRs were detected. The absence of stress-linked 5mC changes suggests distinct fungal epigenetic regulation mechanisms, warranting future study of 5mC/6mA dynamics across rare earth ion concentrations. (*H* = T/A/C).

WGBS of *A. niger* FH1 exposed to 600 mg/L Ce(III) and control (0 mg/L) generated 20.92–28.76 million raw reads, with clean bases (2.45–2.97 Gb) exhibiting Q30 scores >95.00% and bisulfite conversion rates >99.60%. Alignment rates to the reference genome ranged from 41.23 to 80.75% (Supplementary Table 11; Pel et al., 2007). Global DNA methylation levels decreased by 11.11% under Ce(III) (0.32% vs. control: 0.36%), with reductions in CG (13.8%), CHG (11.4%), and CHH (0.08%) contexts (Figure 8A). Methylated cytosines remained predominantly CHH-biased (Ce(III): 49.87%; control: 53.58%), with mCG and mCHG proportions almost unchanged (Figure 8B).

Methylation levels near transcription start sites (TSS) dropped sharply in all sequence contexts under Ce(III) (Figures 8C–E). Gene body regions showed lower CG and CHG methylation compared to flanking regions, mirroring patterns in *Brassica rapa* (Liu et al., 2018). CHH methylation, however, remained uniform across genic and intergenic regions. No significant differentially methylated regions (DMRs) were identified between groups. As reported, treating *Isoetes sinensis* with three different concentrations of Pb(II) and Cd(II) led to concentration - dependent variations in overall DNA methylation. When exposed to high concentrations of Pb(II) (5000 mg/L) and Cd(II) (500 mg/L), no significant differences in DNA methylation levels were observed (CK: 46.96%, Pb: 48.23%, Cd: 48.1%) (Ding et al., 2019). Thus, it is hypothesized that the relatively minor changes in DNA methylation levels may be related to the increased Ce(III) concentrations in the exposure environment. In addition, previous reports have indicated that 6-methyladenine (6mA) was only found in prokaryotes. However, recent studies have shown that 6mA methylation also exists in some fungi, and 6mA methylation can also participate in responding to environmental changes (Mondo et al., 2017). According to the report by Lax et al. (2024), under both light and dark cultivation conditions, the levels of 5mC and 6mA methylation in *Phycomyces* showed no significant changes, whereas the 6mA methylation level of Mucor likewise did not change significantly, but the 5mC methylation level varied in response to light conditions, suggesting that there are mechanisms of methylation of the different types of DNA from different fungi in response to changes in the same environments differences. The lack of involvement of 5mC methylation in regulating genes related to Ce(III) stress response might also explain why no significant differences in 5mC methylation levels were observed under Ce(III) stress. Therefore, to elucidate the relationship between DNA methylation and the regulation of Ce(III) resistance gene expression in *A. niger*, further studies are needed by measuring changes in 5mC and 6mA methylation levels under stress from different concentrations of rare earth ions.

## Conclusion

This work provides a multi-faceted understanding of *A. niger* FH1’s response to Ce(III). We demonstrate its efficient biosorption capability, driven by functional group-mediated chemisorption. More significantly, we elucidate the complex metabolic trade-offs underlying its tolerance: suppression of energy-intensive oxidative phosphorylation is counterbalanced by the upregulation of glycolysis and PPP for ATP and NADPH production. Concurrently, the restructuring of amino acid metabolism bolsters antioxidant defenses (proline, phenolics, GSH pathway precursors) and enhances metal chelation capacity (melanin). This integrated response highlights the sophisticated adaptive machinery of fungi in metal-stressed environments.

Our optimized biosorption system (pH 7.0, 100 mg/L Ce(III), 2000 mg/L biomass) achieved an adsorption capacity of 42.61 mg/g. Kinetic analysis revealed a rapid biphasic adsorption process, well-described by both pseudo-first-order and pseudo-second-order models (R^2^ > 0.99), suggesting an initial physical adsorption phase (likely electrostatic interactions) followed by chemisorption. The superior fit of the Langmuir isotherm (*R*^2^ = 0.9705) over the Freundlich model indicates monolayer adsorption onto homogeneous binding sites. This aligns with previous studies on *A. niger* adsorption of other REEs like Y(III) and Nd(III), reinforcing the role of fungal biomass as efficient biosorbents. Spectroscopic analyses (FTIR, XPS, SEM) provided direct evidence for the involvement of key functional groups (-NH2, -OH, -COOH, - PO3-4) in Ce(III) binding. Ligand exchange and coordination, particularly through amine groups forming Ce-N complexes and carboxyl/hydroxyl groups forming Ce-O bonds, were identified as primary mechanisms. SEM further revealed significant Ce(III)-induced morphological alterations (surface roughening, invagination, aggregation), consistent with a defensive strategy to minimize toxic metal exposure. *A. niger* FH1’s 42.61 mg/g for Ce(III) surpasses many fungal reports for REEs, particularly given its high tolerance (600 mg/L), which exceeds typical thresholds and enables handling of concentrated industrial effluents.

Transcriptomic analysis unveiled a profound reprogramming of *A. niger* FH1 metabolism under Ce(III) stress (600 mg/L), with 3,733 differentially expressed genes (DEGs). Crucially, we observed a marked suppression of oxidative phosphorylation pathways. Downregulation of genes encoding core electron transport chain components (NADH dehydrogenase, cytochrome c reductase, cytochrome c oxidase) and ATP synthase suggests impaired mitochondrial function and reduced ATP synthesis efficiency. This disruption likely represents a primary mechanism of Ce(III) toxicity, potentially driven by Ce(III)-induced ROS damaging the mitochondrial machinery, as reported for other metals like Cd(II) and Cu(II).

Conversely, glycolysis and the pentose phosphate pathway (PPP) were significantly upregulated. Increased expression of genes encoding key enzymes (Hexokinase, GAPDH, Enolase; 6PGDH, Transketolase) points to an adaptive metabolic shift. Enhanced glycolysis provides essential ATP and metabolic intermediates under conditions of mitochondrial dysfunction, while the PPP surge generates crucial NADPH. NADPH is vital for maintaining the cellular redox balance (e.g., regenerating reduced glutathione, GSH) to counteract Ce(III)-induced oxidative stress. This compensatory upregulation of cytosolic energy and reducing power pathways is a critical survival strategy.

Furthermore, amino acid metabolism pathways underwent significant modulation. Upregulation in phenylalanine (increased PAL), tyrosine (increased TYR, HPPD), arginine/proline (increased ARG, P5CR), and glycine/serine/threonine (increased AMT, TA) metabolism serves multiple protective functions: (1) Antioxidant Production: PAL activation promotes phenolic antioxidant synthesis; TYR/HPPD upregulation enhances melanin production, acting as a ROS scavenger and metal chelator.(2) Osmoprotection and ROS Scavenging: ARG and P5CR upregulation drives proline accumulation, a known osmoprotectant and hydroxyl radical scavenger under metal stress.(3) GSH Precursor Supply: AMT and TA upregulation likely increase glycine availability, a key precursor for the synthesis of the major antioxidant glutathione (GSH).

The induction of melanin synthesis via TYR upregulation is particularly noteworthy. Beyond its antioxidant role, melanin provides additional functional groups on the fungal cell wall, enhancing its capacity to adsorb Ce(III) ions, as observed in Cu(II)-stressed *A. niger*. This suggests a dual role for certain metabolic shifts: direct stress mitigation and enhanced metal sequestration.

Interestingly, WGBS revealed only a subtle global decrease in 5mC methylation (11.11%, primarily in CG and CHG contexts) under Ce(III) stress, with no significant Differentially Methylated Regions (DMRs) identified. This contrasts with some studies showing stress-induced methylation changes in other fungi. While the functional significance of this minor shift remains unclear, it suggests that the robust transcriptional response observed in FH1 may be primarily regulated by mechanisms other than 5mC DNA methylation, such as transcription factor activation or histone modifications, under the tested conditions. Further investigation into other epigenetic marks and across a range of Ce(III) concentrations is warranted.

## Data Availability

The original contributions presented in the study are publicly available. This data can be found here: NCBI BioProject, accession PRJNA1331455.

## Footnotes

1
https://www.ncbi.nlm.nih.gov/genbank/

2
https://www.ncbi.nlm.nih.gov/bioproject/PRJNA1331455/

## References

[B1] Andrès Y. Gérente C. (2011). *Microbial biosorption of metals.* Berlin: Springer Netherlands, 179–196.

[B2] Arslan S. Kütük N. (2023). Symbolic regression with feature selection of dye biosorption from an aqueous solution using pumpkin seed husk using evolutionary computation-based automatic programming methods. *Expert Syst. Appl.* 231:120676. 10.1016/j.eswa.2023.120676

[B3] Basu A. Ali S. S. Hossain S. S. Asif M. (2022). A review of the dynamic mathematical modeling of heavy metal removal with the biosorption process. *Processes* 10:1154. 10.3390/pr10061154

[B4] Bergsten-Torralba L. Magalhães D. Giese E. Nascimento C. Pinho J. Buss D. (2020). Toxicity of three rare earth elements, and their combinations to algae, microcrustaceans, and fungi. *Ecotoxicol. Environ. Saf.* 201:110795. 10.1016/j.ecoenv.2020.110795 32544742

[B5] Bertagnolli C. Uhart A. Dupin J. da Silva M. Guibal E. Desbrieres J. (2014). Biosorption of chromium by alginate extraction products from Sargassum filipendula: Investigation of adsorption mechanisms using X-ray photoelectron spectroscopy analysis. *Bioresour. Technol.* 164 264–269. 10.1016/j.biortech.2014.04.103 24862002

[B6] Blackwell K. J. Singleton I. Tobin J. M. (1995). Metal cation uptake by yeast: A review. *Appl. Microbiol. Biotechnol.* 43 579–584. 10.1007/BF00164757 7546600

[B7] Bolger A. M. Lohse M. Usadel B. (2014). Trimmomatic: A flexible trimmer for Illumina sequence data. *Bioinformatics* 30 2114–2120. 10.1093/bioinformatics/btu170 24695404 PMC4103590

[B8] Brown R. Struhs E. Mirkouei A. Raja K. Reed D. (2023). Mixed rare earth metals production from surface soil in Idaho, USA: techno-economic analysis and greenhouse gas emission assessment. *Sci. Total Environ.* 944:173945. 10.1016/j.scitotenv.2024.173945 38876346

[B9] Buayam N. Davey M. P. Smith A. G. Pumas C. (2019). Effects of copper and pH on the growth and physiology of desmodesmus sp. AARLG074. *Metabolites* 9:84. 10.3390/metabo9050084 31052259 PMC6572535

[B10] Cao Y. Shao P. Chen Y. Zhou X. Yang L. Shi H. (2021). A critical review of the recovery of rare earth elements from wastewater by algae for resources recycling technologies. *Resour. Conserv. Recycling* 169:105519. 10.1016/j.resconrec.2021.105519

[B11] Castro-Guerrero N. Rodríguez-Zavala J. Marín-Hernández A. Rodríguez-Enríquez S. Moreno-Sánchez R. (2008). Enhanced alternative oxidase and antioxidant enzymes under Cd(2+) stress in Euglena. *J. Bioenerg. Biomembr.* 40 227–235. 10.1007/s10863-007-9098-6 17899336

[B12] Charlotte Maluleke K. Camagu Goso X. Ndlovu S. Matinde L. (2023). Investigations into the extraction of rare earth elements from Zandkopsdrift ore using the sulfation roasting process. *Minerals Eng.* 191:107902. 10.1016/j.mineng.2022.107902

[B13] Chauhan J. Saini I. Kaushik P. (2020). Studies on the biosorption potential of copper by Rhizopus arrhizus biomass. *bioRxiv [Preprint]* 10.1101/2020.07.13.201566

[B14] Cheng Y. Zhang T. Zhang L. Ke Z. Kovarik L. Dong H. (2016). Resource recovery: Adsorption and biomineralization of cerium by Bacillus licheniformis. *J. Hazard Mater.* 426:127844. 10.1016/j.jhazmat.2021.127844 34838363

[B15] Choe M. Einav T. Phillips R. Titov D. V. (2025). Glycolysis model shows that allostery maintains high ATP and limits accumulation of intermediates. *Biophys. J.* 124 1562–1586. 10.1016/j.bpj.2025.03.037 40186355 PMC12256850

[B16] Cid H. A. Flores M. Pizarro J. Castillo X. Barrios D. (2018). Mechanisms of Cu2+ biosorption on Lessonia nigrescens dead biomass: Functional groups interactions and morphological characterization. *J. Environ. Chem. Eng.* 6 2696–2704. 10.1016/j.jece.2018.03.034

[B17] Clasquin M. F. Melamud E. Singer A. Gooding J. R. Xu X. Dong A. (2011). Riboneogenesis in yeast. *Cell* 145 969–980. 10.1016/j.cell.2011.05.022 21663798 PMC3163394

[B18] Dachuan Y. Jinyu Q. (2021). The physiological response of Ectomycorrhizal fungus Lepista sordida to Cd and Cu stress. *PeerJ* 9:e11115. 10.7717/peerj.11115 33959412 PMC8054734

[B19] Das N. Das D. (2013). Recovery of rare earth metals through biosorption: An overview. *J. Rare Earths* 31 933–943. 10.1016/S1002-0721(13)60009-5

[B20] de Sena Brandine G. Smith A. D. (2019). Falco: High-speed FastQC emulation for quality control of sequencing data. *F1000Res* 8:1874. 10.12688/f1000research.21142.2 33552473 PMC7845152

[B21] Ding G. H. Guo D. D. Guan Y. Chi C. Y. Liu B. D. (2019). Changes of DNA methylation of Isoetes sinensis under Pb and Cd stress. *Environ. Sci. Pollut. Res. Int.* 26 3428–3435. 10.1007/s11356-018-3864-3 30515690

[B22] Doğanlar Z. B. Doğanlar O. Tabakçıoğlu K. (2014). Genotoxic effects of heavy metal mixture in drosophila melanogaster: Expressions of heat shock proteins, RAPD profiles and mitochondrial DNA sequence. *Water Air Soil Pollut.* 225:2104. 10.1007/s11270-014-2104-9

[B23] Eisenman H. C. Casadevall A. (2012). Synthesis and assembly of fungal melanin. *Appl. Microbiol. Biotechnol.* 93 931–940. 10.1007/s00253-011-3777-2 22173481 PMC4318813

[B24] El-Gazzar N. Abdo E. Rabie G. El-Sayed M. T. (2025). Suppression of mycotoxins production and efficient chelation of heavy metals using natural melanin originated from Aspergillus flavus and Aspergillus carbonarius. *BMC Biotechnol.* 25:6. 10.1186/s12896-024-00941-7 39794745 PMC11724575

[B25] Elkomy R. Rizk O. (2019). Bioremoval of copper by marine blue green algae Phormodium formosum and Oscillatoria simplicissima. *Indian J. Sci. Technol.* 12 1–7. 10.17485/ijst/2019/v12i1/134088

[B26] Ezzati R. Azizi M. Ezzati S. A. (2024). Theoretical approach for evaluating the contributions of pseudo-first-order and pseudo-second-order kinetics models in the Langmuir rate equation. *Vacuum* 222:113018. 10.1016/j.vacuum.2024.113018

[B27] Fan B. Zhao L. Feng Z. Liu D. Yin W. Long X. (2021). Leaching behaviors of calcium and magnesium in ion-adsorption rare earth tailings with magnesium sulfate. *Trans. Nonferrous Metals Soc. China* 31 288–296. 10.1016/S1003-6326(21)65495-X

[B28] Fesko K. (2016). Threonine aldolases: Perspectives in engineering and screening the enzymes with enhanced substrate and stereo specificities. *Appl. Microbiol. Biotechnol.* 100 2579–2590. 10.1007/s00253-015-7218-5 26810201 PMC4761611

[B29] Fuentes-Lemus E. Mariotti M. Hägglund P. Leinisch F. Fierro A. Silva E. (2025). Binding of rose bengal to lysozyme modulates photooxidation and cross-linking reactions involving tyrosine and tryptophan. *Free Radic Biol. Med.* 143 375–386. 10.1016/j.freeradbiomed.2019.08.023 31446058

[B30] Gadd G. M. (1994). *The Genus Aspergillus: from taxonomy and genetics to industrial application.* Berklin: Springer, 361–374.

[B31] Giese E. C. Dekker R. F. H. Barbosa-Dekker A. M. (2019). Biosorption of lanthanum and samarium by viable and autoclaved mycelium of Botryosphaeria rhodina MAMB-05. *Biotechnol. Prog.* 35:e2783. 10.1002/btpr.2783 30738002

[B32] Gomaa O. Jassim A. Chanda A. (2022). Bioremoval of PVP-coated silver nanoparticles using Aspergillus niger: The role of exopolysaccharides. *Environ. Sci. Pollut. Res. Int.* 29 31501–31510. 10.1007/s11356-021-18018-9 35001269 PMC8743098

[B33] Gougousi T. Chen Z. (2008). Deposition of yttrium oxide thin films in supercritical carbon dioxide. *Thin Solid Films* 516 6197–6204. 10.1016/j.tsf.2007.11.104

[B34] Guiza S. (2017). Biosorption of heavy metal from aqueous solution using cellulosic waste orange peel. *Ecol. Eng.* 99 134–140. 10.1016/j.ecoleng.2016.11.043

[B35] Gupta N. K. Gupta A. Ramteke P. Sahoo H. Sengupta A. (2019). Biosorption-a green method for the preconcentration of rare earth elements (REEs) from waste solutions: A review. *J. Mol. Liquids* 274 148–164. 10.1016/j.molliq.2018.10.134

[B36] Gupta V. K. Rastogi A. Saini V. K. Jain N. (2006). Biosorption of copper(II) from aqueous solutions by Spirogyra species. *J. Colloid Interface Sci.* 296 59–63. 10.1016/j.jcis.2005.08.033 16168429

[B37] Hermassi M. Granados M. Valderrama C. Ayora C. Cortina J. (2022). Recovery of rare earth elements from acidic mine waters: An unknown secondary resource. *Sci. Total Environ.* 810:152258. 10.1016/j.scitotenv.2021.152258 34896513

[B38] Hisada M. Kawase Y. (2018). Recovery of rare-earth metal neodymium from aqueous solutions by poly-γ-glutamic acid and its sodium salt as biosorbents: Effects of solution pH on neodymium recovery mechanisms. *J. Rare Earths* 36 528–536. 10.1016/j.jre.2018.01.001

[B39] Ho Y. S. (2006). Review of second-order models for adsorption systems. *J. Hazard Mater.* 136 681–689. 10.1016/j.jhazmat.2005.12.043 16460877

[B40] Hyun M. W. Yun Y. H. Kim J. Y. Kim S. H. (2011). Fungal and plant phenylalanine ammonia-lyase. *Mycobiology* 39 257–265. 10.5941/myco.2011.39.4.257 22783113 PMC3385129

[B41] Jamir I. Ezung L. Merry L. Tikendra L. Sanayaima R. Nongdam P. (2024). Heavy metals clean up: The application of fungi for biosorption. *Geomicrobiol. J.* 41 1–12. 10.1080/01490451.2024.2307899

[B42] Jańczak-Pieniążek M. Cichoński J. Michalik P. Chrzanowski G. (2022). Effect of heavy metal stress on phenolic compounds accumulation in winter wheat plants. *Molecules* 28:241. 10.3390/molecules28010241 36615433 PMC9822316

[B43] Jia B. Cui X. Zhang Z. Li X. Hou Y. Luo J. (2024). Arbuscular mycorrhizal fungi regulate amino acid metabolism, phytohormones and glycolysis pathway to promote the growth of Suaeda salsa under combined Cd and NaCl stresses. *Plant Physiol. Biochem.* 214:108921. 10.1016/j.plaphy.2024.108921 38991594

[B44] Kanehisa M. Furumichi M. Sato Y. Ishiguro-Watanabe M. Tanabe M. (2020). KEGG: Integrating viruses and cellular organisms. *Nucleic Acids Res.* 49 D545–D551. 10.1093/nar/gkaa970 33125081 PMC7779016

[B45] Keerthiraju E. Du C. Tucker G. Greetham D. A. (2019). Role for COX20 in tolerance to oxidative stress and programmed cell death in Saccharomyces cerevisiae. *Microorganisms* 7:575. 10.3390/microorganisms7110575 31752220 PMC6920987

[B46] Kierans S. J. Taylor C. T. (2024). Glycolysis: A multifaceted metabolic pathway and signaling hub. *J. Biol. Chem.* 300:107906. 10.1016/j.jbc.2024.107906 39442619 PMC11605472

[B47] Kim D. Paggi J. Park C. Bennett C. Salzberg S. (2019). Graph-based genome alignment and genotyping with HISAT2 and HISAT-genotype. *Nat. Biotechnol.* 37 907–915. 10.1038/s41587-019-0201-4 31375807 PMC7605509

[B48] Klekota J. Roth F. P. Schreiber S. L. (2006). Query Chem: A google-powered web search combining text and chemical structures. *Bioinformatics* 22 1670–1673. 10.1093/bioinformatics/btl155 16672261

[B49] Laporte D. González A. Moenne A. (2020). Copper-induced activation of MAPKs, CDPKs and CaMKs triggers activation of hexokinase and inhibition of pyruvate kinase leading to increased synthesis of ASC, GSH and NADPH in Ulva compressa. *Front. Plant Sci.* 11:990. 10.3389/fpls.2020.00990 32733511 PMC7363978

[B50] Lavín J. L. Oguiza J. A. Ramírez L. Pisabarro A. G. (2008). Comparative genomics of the oxidative phosphorylation system in fungi. *Fungal Genet. Biol.* 45 1248–1256. 10.1016/j.fgb.2008.06.005 18647654

[B51] Lax C. Mondo S. Osorio-Concepción M. Muszewska A. Corrochano-Luque M. Gutiérrez G. (2024). Symmetric and asymmetric DNA N6-adenine methylation regulates different biological responses in Mucorales. *Nat. Commun.* 15:6066. 10.1038/s41467-024-50365-2 39025853 PMC11258239

[B52] Liang B. Gu J. Zeng X. Yuan W. Rao M. Xiao B. (2024). A review of the occurrence and recovery of rare earth elements from electronic waste. *Molecules* 29:4624. 10.3390/molecules29194624 39407554 PMC11477848

[B53] Liang W. Zhang W. Lv Z. Li C. (2020). 4-Hydroxyphenylpyruvate dioxygenase from sea cucumber Apostichopus japonicus negatively regulates reactive oxygen species production. *Fish Shellfish Immunol.* 101 261–268. 10.1016/j.fsi.2020.04.013 32276034

[B54] Liang X. Dickman M. Becker D. (2014). Proline biosynthesis is required for endoplasmic reticulum stress tolerance in Saccharomyces cerevisiae. *J. Biol. Chem.* 289 27794–27806. 10.1074/jbc.M114.562827 25112878 PMC4183814

[B55] Liu G. Xia Y. Liu T. Dai S. Hou X. (2018). The DNA methylome and association of differentially methylated regions with differential gene expression during heat stress in Brassica rapa. *Int. J. Mol. Sci.* 19:1414. 10.3390/ijms19051414 29747401 PMC5983725

[B56] Ma H. Xu X. Zhao X. Liu H. Chen H. (2015). Impacts of drought stress on soluble carbohydrates and respiratory enzymes in fruit body of Auricularia auricula. *Biotechnol. Biotechnol. Equip.* 29 10–14. 10.1080/13102818.2014.984522 26019613 PMC4433945

[B57] Ma J. Li S. Wang J. Jiang S. Panchal B. Sun Y. (2023). Bioleaching rare earth elements from coal fly ash by Aspergillus niger. *Fuel* 354:129387. 10.1016/j.fuel.2023.129387

[B58] Maleke M. Valverde A. Vermeulen J. Cason E. Gomez-Arias A. Moloantoa K. (2019). Biomineralization and bioaccumulation of europium by a thermophilic metal resistant bacterium. *Front. Microbiol.* 10:81. 10.3389/fmicb.2019.00081 30761115 PMC6363818

[B59] Malhotra N. Hsu H. S. Liang S. T. Roldan M. J. M. Lee J. S. Ger T. R. (2020). An updated review of toxicity effect of the rare earth elements (REEs) on aquatic organisms. *Animals* 10:1663. 10.3390/ani10091663 32947815 PMC7552131

[B60] Mansoor S. Ali A. Kour N. Bornhorst J. AlHarbi K. Rinklebe J. (2023). Heavy metal induced oxidative stress mitigation and ROS scavenging in plants. *Plants* 12:3003. 10.3390/plants12163003 37631213 PMC10459657

[B61] Martinez R. E. Pourret O. Takahashi Y. (2014). Modeling of rare earth element sorption to the gram positive Bacillus subtilis bacteria surface. *J. Colloid Interface Sci.* 413 106–111. 10.1016/j.jcis.2013.09.037 24183437

[B62] McGraw K. J. (2003). Melanins, metals, and mate quality. *Oikos* 102 402–406. 10.1034/j.1600-0579.2003.12513.x

[B63] Meng X. Zhao H. Zhang Y. Shen L. Gu S. Qiu Z. (2022). Simulated bioleaching of ion-adsorption rare earth ore using metabolites of biosynthetic citrate: An alternative to cation exchange leaching. *Minerals Eng.* 189:107900. 10.1016/j.mineng.2022.107900

[B64] Mohammadi Alagoz S. Hadi H. Toorchi M. Pawłowski T. Asgari Lajayer B. Price G. (2023). Morpho-physiological responses and growth indices of triticale to drought and salt stresses. *Sci. Rep.* 13:8896. 10.1038/s41598-023-36119-y 37264097 PMC10235095

[B65] Mondo S. J. Dannebaum R. O. Kuo R. C. Louie K. B. Bewick A. J. LaButti K. (2017). Widespread adenine N6-methylation of active genes in fungi. *Nat. Genet.* 49 964–968. 10.1038/ng.3859 28481340

[B66] Mukherjee A. Das D. Kumar Mondal S. Biswas R. Kumar Das T Boujedaini N. (2010). Tolerance of arsenate-induced stress in Aspergillus niger, a possible candidate for bioremediation. *Ecotoxicol. Environ. Saf.* 73 172–182. 10.1016/j.ecoenv.2009.09.015 19811831

[B67] Obregón-Castro C. Prudencio M. Diamantion C. Carvalho E. Russo D. Marques R. (2023). Geochemical behaviour of rare earth elements throughout an acid mine drainage passive treatment system in the lousal mine area, portugal. *Mine Water Environ.* 42 533–545. 10.1007/s10230-023-00954-2

[B68] Okay S. Yildirim V. Büttner K. Becher D. Özcengiz G. (2020). Dynamic proteomic analysis of Phanerochaete chrysosporium under copper stress. *Ecotoxicol. Environ. Saf.* 198:110694. 10.1016/j.ecoenv.2020.110694 32388186

[B69] Oliveira R. C. Hammer P. Guibal E. Taulemesse J.-M. Garcia O. (2014). Characterization of metal–biomass interactions in the lanthanum(III) biosorption on Sargassum sp. using SEM/EDX, FTIR, and XPS: Preliminary studies. *Chem. Eng. J.* 239 381–391. 10.1016/j.cej.2013.11.042

[B70] Panda S. Costa R. Shah S. Mishra S. Bevilaqua D. Akcil A. (2021). Biotechnological trends and market impact on the recovery of rare earth elements from bauxite residue (red mud) – A review. *Resour. Conserv. Recycling* 171:105645. 10.1016/j.resconrec.2021.105645

[B71] Paper M. Jung P. Koch M. Lakatos M. Nilges T. Brück T. (2023). Stripped: contribution of cyanobacterial extracellular polymeric substances to the adsorption of rare earth elements from aqueous solutions. *Front. Bioeng. Biotechnol.* 11:1299349. 10.3389/fbioe.2023.1299349 38173874 PMC10762542

[B72] Park D. M. Reed D. W. Yung M. C. Eslamimanesh A. Lencka M. M. Anderko A. (2016). Bioadsorption of rare earth elements through cell surface display of lanthanide binding tags. *Environ. Sci. Technol.* 50 2735–2742. 10.1021/acs.est.5b06129 26836847 PMC5381720

[B73] Peelman S. Sun Z. H. I. Sietsma J. Yang Y. (2016). *Rare earths industry.* Amsterdam: Elsevier, 319–334.

[B74] Pel H. de Winde J. Archer D. Dyer P. Hofmann G. Schaap P. (2007). Genome sequencing and analysis of the versatile cell factory Aspergillus niger CBS 513.88. *Nat. Biotechnol.* 25 221–231. 10.1038/nbt1282 17259976

[B75] Perdigão Cota de Almeida S. Rozas E. E. Oller do Nascimento C. A. Dias M. Mendes M. A. (2021). Metabolomic and secretomic approach to the resistance features of the fungus Aspergillus niger IOC 4687 to copper stress. *Metallomics* 13:mfaa010. 10.1093/mtomcs/mfaa010 33570139

[B76] Priyanka Y. Dwivedi S. K. (2023). Fungi mediated detoxification of heavy metals: Insights on mechanisms, influencing factors and recent developments. *J. Water Process Eng.* 53:103800. 10.1016/j.jwpe.2023.103800

[B77] Pulido-Reyes G. Rodea-Palomares I. Das S. Sakthivel T. S. Leganes F. Rosal R. (2015). Untangling the biological effects of cerium oxide nanoparticles: The role of surface valence states. *Sci. Rep.* 5:15613. 10.1038/srep15613 26489858 PMC4615008

[B78] Ramrakhiani L. Halder A. Majumder A. Mandal A. Majumder M. Ghosh S. (2017). Industrial waste derived biosorbent for toxic metal remediation: Mechanism studies and spent biosorbent management. *Chem. Eng. J.* 308 1048–1064. 10.1016/j.cej.2016.09.145

[B79] Revel M. van Drimmelen C. K. E. Weltje L. Hursthouse A. Heise S. (2025). Effects of rare earth elements in the aquatic environment: Implications for ecotoxicological testing. *Crit. Rev. Environ. Sci. Technol.* 55 334–375. 10.1080/10643389.2024.2406992

[B80] Reyes J. S. Cortés-Ríos J. Fuentes-Lemus E. Rodriguez-Fernandez M. Davies M. J. López-Alarcón C. (2024). Competitive oxidation of key pentose phosphate pathway enzymes modulates the fate of intermediates and NAPDH production. *Free Radic. Biol. Med.* 222 505–518. 10.1016/j.freeradbiomed.2024.05.050 38848786

[B81] Rezk M. M. Morse W. M. (2023). The highly tolerant fungi and extraction potentiality of lanthanum: Application on rare earth elements concentrate derivative from monazite. *Toxicol. Environ. Health Sci.* 15 31–39. 10.1007/s13530-022-00155-4

[B82] Roozegar M. Behnam S. D. E. (2018). An eco-friendly approach for copper (II) biosorption on alga Cystoseira indica and its characterization. *Environ. Progress Sustainable Energy.* 38 S323–S330. 10.1002/ep.13044

[B83] Rutter J. Hughes A. L. (2015). Power(2): The power of yeast genetics applied to the powerhouse of the cell. *Trends Endocrinol. Metab.* 26 59–68. 10.1016/j.tem.2014.12.002 25591985 PMC4315768

[B84] Ruzafa C. Solano F. Sanchez-Amat A. (1994). The protein encoded by the Shewanella colwelliana melA gene is a p-hydroxyphenylpyruvate dioxygenase. *FEMS Microbiol. Lett.* 124 179–184. 10.1111/j.1574-6968.1994.tb07282.x 7813886

[B85] Shah S. S. Palmieri M. C. Sponchiado S. R. P. Bevilaqua D. (2020). Enhanced bio-recovery of aluminum from low-grade bauxite using adapted fungal strains. *Braz. J. Microbiol.* 51 1909–1918. 10.1007/s42770-020-00342-w 32748245 PMC7688833

[B86] Shahhosseini M. Doulati Ardejani F. Baafi E. (2017). Geochemistry of rare earth elements in a neutral mine drainage environment, Anjir Tangeh, northern Iran. *Int. J. Coal Geol.* 183 120–135. 10.1016/j.coal.2017.10.004

[B87] Sharma J. Kumar S. Singh P. Kumar V. Verma S. Khyalia P. (2024). Emerging role of osmoprotectant glycine betaine to mitigate heavy metals toxicity in plants: A systematic review. *Biol. Futur.* 75 159–176. 10.1007/s42977-023-00198-9 38183566

[B88] Shin D. Kim J. Kim B.-S. Jeong J. Lee J.-C. (2015). Use of phosphate solubilizing bacteria to leach rare earth elements from monazite-bearing ore. *Minerals* 5 189–202. 10.3390/min5020189

[B89] Siddiqi K. S. Husen A. (2011). Significance of brassinosteroids and their derivatives in the development and protection of plants under abiotic stress. *Biologia* 76 2837–2857. 10.1007/s11756-021-00853-3

[B90] Signorelli S. Coitiño E. L. Borsani O. Monza J. (2014). Molecular mechanisms for the reaction between (^⋅^)OH radicals and proline: Insights on the role as reactive oxygen species scavenger in plant stress. *J. Phys. Chem. B* 118 37–47. 10.1021/jp407773u 24328335

[B91] Słominski A. Moellmann G. Kuklinska E. Bomirski A. Pawelek J. (1988). Positive regulation of melanin pigmentation by two key substrates of the melanogenic pathway, L-tyrosine and L-dopa. *J. Cell Sci.* 89 287–296. 10.1242/jcs.89.3.287 3143738

[B92] Takahashi Y. Châtellier X. Hattori K. H. Kato K. Fortin D. (2005). Adsorption of rare earth elements onto bacterial cell walls and its implication for REE sorption onto natural microbial mats. *Chem. Geol.* 219 53–67. 10.1016/j.chemgeo.2005.02.009

[B93] The Gene Ontology Consortium. (2020). The gene ontology resource: Enriching a GOld mine. *Nucleic Acids Res.* 49 D325–D334. 10.1093/nar/gkaa1113 33290552 PMC7779012

[B94] Tian Y. Peng K. Bao Y. Zhang D. Meng J. Wang D. (2021). Glucose-6-phosphate dehydrogenase and 6-phosphogluconate dehydrogenase genes of winter wheat enhance the cold tolerance of transgenic Arabidopsis. *Plant Physiol. Biochem.* 161 86–97. 10.1016/j.plaphy.2021.02.005 33581622

[B95] Timm S. Giese J. Engel N. Wittmiß M. Florian A. Fernie A. R. (2018). T-protein is present in large excess over the other proteins of the glycine cleavage system in leaves of Arabidopsis. *Planta* 247 41–51. 10.1007/s00425-017-2767-8 28866761

[B96] Vapnik H. Kim H. Kim Y. Ooi A. Vibbert H. Park A. (2025). Selective electrochemical recovery of cerium over lanthanum from complex waste feedstocks by alternating current electro-precipitation. *Chem. Eng. J.* 504:158537. 10.1016/j.cej.2024.158537

[B97] Vescovi M. Zaffagnini M. Festa M. Trost P. Lo Schiavo F. Costa A. (2013). Nuclear accumulation of cytosolic glyceraldehyde-3-phosphate dehydrogenase in cadmium-stressed Arabidopsis roots. *Plant Physiol.* 162 333–346. 10.1104/pp.113.215194 23569110 PMC3641213

[B98] Wang F. Wang W. Zhu Y. Wang A. (2017). Evaluation of Ce(III) and Gd(III) adsorption from aqueous solution using CTS-g-(AA-co-SS)/ISC hybrid hydrogel adsorbent. *J. Rare Earths* 35 697–708. 10.1016/S1002-0721(17)60966-9

[B99] Wang H. Zhang X. Wang L. Zhu B. Guo W. Liu W. (2020). Biochemical responses and DNA damage induced by herbicide QYR301 in earthworm (Eisenia fetida). *Chemosphere* 244:125512. 10.1016/j.chemosphere.2019.125512 31816546

[B100] Wang L. Liao C. Yang Y. Xu H. Xiao H. Yan C. (2017). Effects of organic acids on the leaching process of ion-adsorption type rare earth ore. *J. Rare Earths* 35 1233–1238. 10.1016/j.jre.2017.07.001

[B101] Wang Q. Xu Y. Zhang M. Zhu F. Sun M. Lian X. (2021). Transcriptome and metabolome analysis of stress tolerance to aluminium in Vitis quinquangularis. *Planta* 254:105. 10.1007/s00425-021-03759-1 34687358

[B102] Wang S.-T. (2021). Oxidative phosphorylation system as the target of glycinin basic peptide against Aspergillus niger. *LWT* 150:111977. 10.1016/j.lwt.2021.111977

[B103] Wang W. Xu C. Jin Y. Zhang Z. Yan R. Zhu D. (2020). The accumulation of rare-earth yttrium ions by Penicillium sp. ZD28. *AMB Express* 10:25. 10.1186/s13568-020-0961-8 32016669 PMC6997312

[B104] Wang Y. Wang D. Wei G. Shao N. (2022). Enhanced co-production of S-adenosylmethionine and glutathione by an ATP-oriented amino acid addition strategy. *Bioresour. Technol.* 107 19–24. 10.1016/j.biortech.2011.12.030 22244899

[B105] Wierzba S. (2017). Biosorption of nickel (II) and zinc (II) from aqueous solutions by the biomass of yeast Yarrowia lipolytica. *Green Sci.* 19 1–10. 10.1515/pjct-2017-0001

[B106] Williamson D. J. Webb M. E. Turnbull W. B. (2014). Depsipeptide substrates for sortase-mediated N-terminal protein ligation. *Nat. Protoc.* 9 253–262. 10.1038/nprot.2014.003 24407354

[B107] Xi Y. Li W. (2009). BSMAP: Whole genome bisulfite sequence MAPping program. *BMC Bioinformatics* 10:232. 10.1186/1471-2105-10-232 19635165 PMC2724425

[B108] Xu J. Chen G. L. Sun X. Z. Fan X. W. You-Zhi L. (2015). Paths and determinants for Penicillium janthinellum to resist low and high copper. *Sci. Rep.* 5:10590. 10.1038/srep10590 26265593 PMC4642507

[B109] Xu J. Li L. Wang H. Gao Z. Wang C. Sun R. (2022). Adsorption characteristics of indigenous chromium-resistant Aspergillus niger strain isolated from red soil for remediation of toxic chromium in red soil environments. *Toxics* 11:31. 10.3390/toxics11010031 36668757 PMC9866775

[B110] Yetis Ü. Ozcengiz G. Dilek F. Ergen N. Erbay A. Dolek A. (1998). Heavy metal biosorption by white-rot fungi. *Water Sci. Technol.* 38 323–330. 10.1016/S0273-1223(98)00515-0

[B111] Young K. Tscherner A. Zhang B. Meredith M. McClatchie T. Trasler J. (2022). 5,10-Methylenetetrahydrofolate reductase becomes phosphorylated during meiotic maturation in mouse oocytes. *Zygote* 30 674–688. 10.1017/S0967199422000156 35652653

[B112] Yu G. Wang L.-G. Han Y. He Q.-Y. (2012). clusterProfiler: An R package for comparing biological themes among gene clusters. *OMICS J. Integr. Biol.* 16 284–287. 10.1089/omi.2011.0118 22455463 PMC3339379

[B113] Zhang Y. Su B. Shao S. Li N. Jiao H. Dan Y. (2023). Geochemical behavior and source analysis of rare earth elements in intensive agriculture soils through high-resolution sampling. *Sci. Total Environ.* 905:167777. 10.1016/j.scitotenv.2023.167777 37848147

[B114] Zhao L. Zhu Y. Wang M. Han Y. Xu J. Feng W. (2023). Enolase, a cadmium resistance related protein from hyperaccumulator plant Phytolacca americana, increase the tolerance of *Escherichia coli* to cadmium stress. *Int. J. Phytoremediation.* 25 562–571. 10.1080/15226514.2022.2092064 35802034

[B115] Zhou H. Wang J. Shao S. Yu X. Kang J. Qiu Z. (2024). Comparison of biosorption behavior and mechanism of La3+, Sm3+, Y3+, Nd3+, Er3+ by Aspergillus niger and Bacillus sp. *J. Water Process Eng.* 59 104965. 10.1016/j.jwpe.2024.104965

[B116] Zinicovscaia I. Yushin N. Grozdov D. Peshkova A. Vergel K. Rodlovskaya E. (2009). The remediation of dysprosium-containing effluents using Cyanobacteria spirulina platensis and yeast Saccharomyces cerevisiae. *Microorganisms* 11:2009. 10.3390/microorganisms11082009 37630569 PMC10458459

