# Supplementary material for: Unraveling the stress response and biosorption mechanisms of Aspergillus niger to rare earth element cerium(III) based on transcriptomics and DNA methylomics
Source: Front Microbiol. 2025 Oct 27;16:1674444. doi: 10.3389/fmicb.2025.1674444 (PMC12597938; doi:10.3389/fmicb.2025.1674444)
Supplement: Supplementary file 1 [file Table_1.docx]

**Supplementary materials for the manuscript “Unraveling the Stress Response and Biosorption Mechanisms of *Aspergillus niger* to Rare Earth Element Cerium(III) Based on Transcriptomics and DNA Methylomics”**

Table S1 Parameters of pseudo-first-order and pseudo-second-order kinetic models for biosorption by *A. niger* FH1

| Fungi | Pseudo-first-order model | | |  | Pseudo-second-order model | | |
| --- | --- | --- | --- | --- | --- | --- | --- |
|  | *q_e_* | *K_1_* | *R^2^* |  | *q_e_* | *K_2_* | *R^2^* |
| *A. niger* FH1 | 37.2904 | 0.1445 | 0.9980 |  | 38.4583 | 0.0117 | 0.9968 |

Table S2 Isotherm parameters for biosorption by *A. niger* FH1

| Fungi | Langmuir model | | |  | Freundlich model | | |
| --- | --- | --- | --- | --- | --- | --- | --- |
|  | *q_e_* | *K_L_* | *R_L_^2^* |  | *n* | *K_F_* | *R_F_^2^* |
| *A. niger* FH1 | 46.7875 | 0.0152 | 0.9705 |  | 2.5495 | 4.2756 | 0.9579 |

Table S3 Summary of RNA-seq data statistics

| Sample name | Ce_1 | Ce_2 | Ce_3 | Control_1 | Control_2 | Control_3 |
| --- | --- | --- | --- | --- | --- | --- |
| Raw reads(M) | 61.03 | 68.04 | 72.15 | 49.85 | 48.73 | 49.16 |
| Clean reads(M) | 60.88 | 67.90 | 71.97 | 49.64 | 48.55 | 48.98 |
| Clean_ratio(%) | 99.74% | 99.79% | 99.75% | 99.57% | 99.64% | 99.62% |
| Clean Bases(G) | 8.55 | 9.57 | 10.10 | 7.47 | 8.40 | 8.47 |
| Q30(%) | 95.96% | 96.37% | 96.04% | 93.59% | 94.40% | 95.12% |
| Mapped reads(M) | 55.44 | 61.90 | 65.46 | 42.55 | 42.05 | 44.12 |
| Unique Mapping rate(%) | 91.08% | 91.15% | 90.96% | 90.20% | 89.84% | 90.08% |

Table S4 DEGs related to oxidative phosphorylation under Ce(III) stress

| KEGG Pathway | Gene ID | Description | log_2_(Fold Change) |
| --- | --- | --- | --- |
| Oxidative phosphorylation  (ko00190) | *An01g04630* | ATP synthase | -1.14 |
|  | *An01g04930* | ATP synthase | -1.38 |
|  | *An12g04950* | ATP synthase | -1.32 |
|  | *An16g08550* | ATP synthase | -2.12 |
|  | *An01g10880* | ATP synthase | -2.11 |
|  | *An07g06560* | ATP synthase | -1.58 |
|  | *An14g00820* | ATP synthase | -2.04 |
|  | *An02g01830* | Cytochrome c | -1.97 |
|  | *An14g04170* | Cytochrome c oxidase COX4 | -1.74 |
|  | *An02g09930* | Cytochrome c oxidase COX5A | -1.21 |
|  | *An07g07390* | Cytochrome c oxidase COX5B | -2.09 |
|  | *An11g10200* | Cytochrome c oxidase COX6A | -2.06 |
|  | *An02g01720* | Cytochrome c oxidase COX6B | -2.46 |
|  | *An09g03990* | Cytochrome c oxidase COX7A | -2.54 |
|  | *An04g01560* | Cytochrome c oxidase COX7C | -1.83 |
|  | *An02g04330* | Cytochrome c oxidase COX11 | -2.32 |
|  | *An11g02430* | Cytochrome c oxidase COX15 | -1.88 |
|  | *An02g12620* | Cytochrome c oxidase COX17 | -3.05 |
|  | *An14g04080* | Cytochrome c reductase ISP | -1.16 |
|  | *An01g06180* | Cytochrome c reductase Cyt1 | -1.31 |
|  | *An09g06650* | Cytochrome c reductase QCR2 | -1.17 |
|  | *An04g05220* | Cytochrome c reductase QCR6 | -1.74 |
|  | *An04g01200* | Cytochrome c reductase QCR7 | -1.91 |
|  | *An08g06550* | Cytochrome c reductase QCR8 | -2.39 |
|  | *An11g06200* | NADH dehydrogenase Ndufs3 | -1.12 |
|  | *An04g00060* | NADH dehydrogenase Ndufs7 | -1.72 |
|  | *An18g05670* | NADH dehydrogenase Ndufs8 | -1.13 |
|  | *An04g05640* | NADH dehydrogenase Ndufv1 | -1.05 |
|  | *An12g04780* | NADH dehydrogenase Ndufv2 | -1.1 |

Table S5 DEGs associated with glycolysis under Ce(III) stress

| KEGG Pathway | Gene ID | Description | log_2_(Fold Change) |
| --- | --- | --- | --- |
| Glycolysis /Gluconeogenesis  (ko00010) | *An01g01590* | Uncharacterized protein | 2.12 |
|  | *An01g12170* | Alcohol dehydrogenase | 1.33 |
|  | *An02g02920* | Triosephosphate isomerase | 1.4 |
|  | *An02g07470* | Fructose-bisphosphate aldolase | 1.98 |
|  | *An02g07650* | Posphoglucomutase | 1.95 |
|  | *An02g09090* | Aldose 1-epimerase | 1.29 |
|  | *An02g14380* | Hexokinase | 1.63 |
|  | *An04g03400* | Aldehyde dehydrogenase | 1.71 |
|  | *An04g05300* | Fructose-1,6-bisphosphatase | 2.03 |
|  | *An08g02260* | Phosphoglycerate kinase | 1.54 |
|  | *An08g07290* | Aldehyde dehydrogenase | 2.34 |
|  | *An08g09750* | Alcohol dehydrogenase | 3.15 |
|  | *An10g00510* | Alcohol dehydrogenase | 1.36 |
|  | *An11g02550* | Phosphoenolpyruvate carboxykinase (ATP) | 2.51 |
|  | *An11g04150* | Alcohol dehydrogenase | 4.66 |
|  | *An13g00950* | Alcohol dehydrogenase | 2.69 |
|  | *An13g03320* | Pyruvate decarboxylase | 4.92 |
|  | *An13g03330* | Uncharacterized protein | 11.94 |
|  | *An14g04410* | Fructose-bisphosphate aldolase | 2.52 |
|  | *An16g01830* | Glyceraldehyde-3-phosphate dehydrogenase | 1.09 |
|  | *An16g05420* | Glucose-6-phosphate isomerase | 1.49 |
|  | *An18g06250* | Enolase | 1.22 |

Table S6 DEGs involved in the pentose phosphate pathway under Ce(III) stress

| KEGG Pathway | Gene ID | Description | log_2_(Fold Change) |
| --- | --- | --- | --- |
| Pentose phosphate pathway  (ko00030) | *An01g07300* | Gluconokinase | 1.24 |
|  | *An02g00250* | Deoxyribose-phosphate aldolase | 2.17 |
|  | *An02g02930* | Ribose 5-phosphate isomerase | 2.59 |
|  | *An02g07470* | Fructose-bisphosphate aldolase | 1.98 |
|  | *An02g07650* | Phosphoglucomutase | 1.95 |
|  | *An04g05300* | Fructose-1,6-bisphosphatase I | 2.03 |
|  | *An08g06570* | Transketolase | 1.69 |
|  | *An11g06120* | 6-phosphogluconate dehydrogenase | 2.56 |
|  | *An14g04410* | Fructose-bisphosphate aldolase | 2.52 |
|  | *An16g05420* | Glucose-6-phosphate isomerase | 1.49 |

Table S7 DEGs linked to phenylalanine metabolism under Ce(III) stress

| KEGG Pathway | Gene ID | Description | log_2_(Fold Change) |
| --- | --- | --- | --- |
| Phenylalanine metabolism  (ko00360) | *An02g00030* | Aromatic amino acid aminotransferase | 3.41 |
|  | *An02g10920* | Primary-amine oxidase | 2.76 |
|  | *An04g01280* | 4-hydroxyphenylpyruvate dioxygenase | 2.18 |
|  | *An07g06400* | Primary-amine oxidase | 4.26 |
|  | *An08g07740* | Phenylalanine ammonia-lyase | 2.74 |
|  | *An12g10150* | Monoamine oxidase | 3.27 |
|  | *An17g00010* | Primary-amine oxidase | 2.98 |
|  | *An01g01830* | Catalase-peroxidase | 2.73 |
|  | *An01g01840* | Monoamine oxidase | 1.62 |
|  | *An03g05880* | Amidase | 2.14 |
|  | *An04g03400* | Uncharacterized protein | 1.71 |
|  | *An07g01900* | 4-hydroxyphenylpyruvate dioxygenase | 1.64 |
|  | *An11g02980* | Amidase | 1.21 |
|  | *An15g00290* | Amidase | 1.23 |
|  | *An16g07500* | Amidase | 2.83 |

Table S8 DEGs related to arginine and proline metabolism under Ce(III) stress

| KEGG Pathway | Gene ID | | Description | log_2_(Fold Change) |
| --- | --- | --- | --- | --- |
| Arginine and proline metabolism (ko00330) | | *An01g01520* | Pyrroline-5-carboxylate Reductase | 3.86 |
|  |  | *An01g15140* | D-arginase | 3.65 |
|  |  | *An02g02290* | 1-pyrroline-5-carboxylate dehydrogenase | 7.06 |
|  |  | *An02g07250* | Arginase | 1.50 |
|  |  | *An04g04130* | Ornithine-oxo-acid transaminase | 1.85 |
|  |  | *An12g10150* | Monoamine oxidase | 3.27 |
|  |  | *An14g01190* | Arginase | 3.08 |
|  |  | *An01g01840* | Monoamine oxidase | 1.62 |
|  |  | *An03g05880* | Amidase | 2.14 |
|  |  | *An04g02420* | Uncharacterized protein | 2.77 |
|  |  | *An07g05000* | 1-pyrroline-5-carboxylate dehydrogenase | 1.23 |
|  |  | *An08g07290* | Aldehyde dehydrogenase | 2.34 |
|  |  | *An09g02560* | Polyamine oxidase | 1.12 |
|  |  | *An10g00330* | 4-hydroxy-2-oxoglutarate aldolase | 2.57 |
|  |  | *An11g02980* | Amidase | 1.21 |
|  |  | *An11g06140* | 1-pyrroline-5-carboxylate dehydrogenase | 1.47 |
|  |  | *An15g00290* | Amidase | 1.23 |
|  |  | *An16g07500* | Amidase | 2.83 |
|  |  | *An16g07700* | 4-hydroxy-2-oxoglutarate aldolase | 2.31 |

Table S9 DEGs associated with glycine, serine, and threonine metabolism under Ce(III) stress

| KEGG Pathway | Gene ID | Description | log_2_(Fold Change) |
| --- | --- | --- | --- |
| Glycine, serine and threonine metabolism  (ko00360) | *An02g10920* | Primary-amine oxidase | 2.76 |
|  | *An02g11560* | Uncharacterized protein | 2.2 |
|  | *An04g09820* | Sarcosine oxidase | 1.48 |
|  | *An07g06400* | Primary-amine oxidase | 4.26 |
|  | *An12g10150* | Monoamine oxidase | 3.27 |
|  | *An14g01470* | D-serine ammonia-lyase | 1.78 |
|  | *An16g02500* | Tryptophan synthase | 2.18 |
|  | *An17g00010* | Primary-amine oxidase | 2.98 |
|  | *An01g01840* | Monoamine oxidase | 1.62 |
|  | *An04g02220* | L-serine ammonia-lyase | 2.97 |
|  | *An05g02380* | Uncharacterized protein | 11.04 |
|  | *An07g00680* | Threonine aldolase | 1.54 |
|  | *An08g03070* | Glycine cleavage system T protein | 1.79 |
|  | *An14g01150* | Glycine Decarboxylase | 1.68 |
|  | *An16g08720* | Cystathionine gamma-lyase | 1.23 |

Table S10 DEGs involved in tyrosine metabolism under Ce(III) stress

| KEGG Pathway | Gene ID | | Description | log_2_(Fold Change) |  |
| --- | --- | --- | --- | --- | --- |
| Tyrosine metabolism (ko00350) | | *An02g00030* | 2-aminoadipate transaminase | 3.41 | |
|  |  | *An02g10920* | Primary-amine oxidase | 2.76 | |
|  |  | *An03g02490* | 4-hydroxy-2-oxoheptanedioate aldolase | 2.94 | |
|  |  | *An04g01280* | 4-hydroxyphenylpyruvate dioxygenase | 2.18 | |
|  |  | *An07g06400* | Primary-amine oxidase | 4.26 | |
|  |  | *An09g02980* | Tyrosinase | 5.05 | |
|  |  | *An09g05130* | Tyrosinase | 5.56 | |
|  |  | *An11g04150* | Alcohol dehydrogenase | 4.66 | |
|  |  | *An12g10150* | Primary-amine oxidase | 3.27 | |
|  |  | *An15g07670* | Tyrosinase | 4.96 | |
|  |  | *An17g00010* | Primary-amine oxidase | 2.98 | |
|  |  | *An01g01840* | Primary-amine oxidase | 1.62 | |
|  |  | *An01g02960* | 1,2-dioxygenase | 2.34 | |
|  |  | *An01g12170* | Alcohol dehydrogenase | 1.33 | |
|  |  | *An07g01900* | hydroxyphenylpyruvate dioxygenase | 1.64 | |
|  |  | *An08g09750* | Alcohol dehydrogenase | 3.15 | |
|  |  | *An09g02440* | 4-hydroxy-2-oxoheptanedioate aldolase | 4.06 | |
|  |  | *An10g00510* | Alcohol dehydrogenase | 1.36 | |
|  |  | *An11g00430* | 1,2-dioxygenase | 1.11 | |
|  |  | *An11g02160* | Maleylacetoacetate isomerase | 1.98 | |
|  |  | *An13g00950* | Alcohol dehydrogenase | 2.69 | |
|  |  | *An17g00200* | Catechol O-methyltransferase | 1.71 | |
|  |  | *An18g01020* | 1,2-dioxygenase | 1.31 | |

Table S11 Summary of WGBS data statistics

| Sample name | Ce_1 | Ce_2 | Ce_3 | Control_1 | Control_2 | Control_3 |
| --- | --- | --- | --- | --- | --- | --- |
| Raw reads(M) | 28.76 | 26.00 | 24.45 | 21.58 | 21.31 | 20.92 |
| Clean reads(M) | 27.17 | 24.26 | 25.94 | 21.31 | 21.02 | 21.22 |
| Clean_ratio(%) | 94.46% | 93.29% | 94.26% | 98.74% | 98.64% | 98.57% |
| Clean Bases(G) | 2.87 | 2.45 | 2.63 | 2.97 | 2.97 | 2.97 |
| Q30(%) | 96.17% | 95.48% | 95.82% | 95.75% | 95.53% | 95.61% |
| GC Content(%) | 34.91% | 34.59% | 34.62% | 29.22% | 28.47% | 28.93% |
| BS Conversion Rate(%) | 99.64% | 99.68% | 99.66% | 99.62% | 99.66% | 99.64% |
| Mapped reads(M) | 11.30 | 10.07 | 10.69 | 17.21 | 15.69 | 16.64 |
| Unique Mapping rate(%) | 41.59% | 41.54% | 41.23% | 80.75% | 74.66% | 78.42% |
| Duplication rate(%) | 5.10% | 4.55% | 4.22% | 13.87% | 11.51% | 12.61% |

Table S12 Genes and primers validated by RT-qPCR

| Gene ID | Primer sequence | |
| --- | --- | --- |
|  | Forward primer (5'–3') | Reverse primer (5'–3') |
| *An01g02270* | CTATACAGCTGGTGGAGATAC | GGAGGCAGATTAGGTCTCTAAC |
| *An01g07680* | CGGAGTAATAACTCCG | GTTGGCAGGCTCACATTCTG |
| *An01g14740* | CGGATTCCACAACACTACCG | CAGAAGGTCCCACACATC |
| *An02g14960* | CTATTATTGCGACGGAG | TGTCAATCTAGCCTTTGAG |
| *An03g00680* | CTTCTCCAACTTCGGCAAC | GAATCGCAGGCCACTTTG |
| *An03g06200* | CACAATGACCTCTAGGAAGCTTC | GTACACCATGTATGAAGCGG |
| *An12g06480* | CGACATGTTCTAACTCTTCC | GAATCCTCGGCCGTATGGAAG |
| *An04g00710* | CGAACTGGACGGTACTC | CGAGCATTTTGCTAGCGG |
| *An07g01150* | CTCAGTACAATACTAACCGC | CCAGGTCGTGGAATATCTG |
| *An08g11590* | GTAATTCCCTACCAGGAAC | CACTGCGTTGATTTGGC |
| *An09g00560* | CAAATCAGCACCCAGGAAC | CAAGGAAGACCACGTTC |
| *An11g05580* | CCTACAGGATCAGACCAAC | CATTATCGCCGTAGAGCTG |
| *An11g08820* | GATGCAGAACCGGCCATTC | CGACGAATGAAAAAGCATCTC |
| *An12g02660* | CATCGCTCCTACATTCAAGC | GAGAAATCTCGGATGAC |
| *An12g02760* | GTACCGTAACGTTAGTAG | CTGGACCACGAACATCTAG |
| *An12g06480* | GAGACGTGGAAGGCACACTAC | CGGTATGGTACAGTGCCAG |
| *An13g03320* | CATTGCTGACACTGGCGATAG | GTATGGTTCGTTGGTCTG |
| *An14g01160* | GACCTTTGTCTTCATGGTTG | CACGGTCTCGGTTAATG |
| *An14g05340* | CTGGTGGATCTACTTGAAC | CATCCAGTTTCCTCATCAG |
| *An18g01470* | GCAATTTCACGAGAGGCCG | GAAACGGTTCCAATGTTCTTG |
| *An18g01780* | CCACTTCATTCATGGATCCCG | CACCCAGTTGGTCACTGTCAC |

The RT-qPCR validation assay comprises three parts: RNA extraction, cDNA synthesis, and RT-qPCR detection; for cDNA synthesis, first, a 16 μL system for gDNA removal is prepared, containing 1 μg template RNA, 4 μL 4×gDNAwipe Mix, and DEPC-treated ddH2O to make up the volume, which is mixed well and incubated at 42 °C for 2 min, and subsequently, a 20 μL reverse transcription (RT) reaction system is prepared with 16 μL of the aforementioned gDNA-removed reaction solution and 4 μL 5×HiScript II qRT SuperMix II, mixed well and subjected to RT on a PCR thermocycler with the program of 50 °C for 5 min followed by 85 °C for 5 s, and the RT product is stored at -20 °C after the reaction; for RT-qPCR detection, upregulated and downregulated differentially expressed genes under Ce(Ⅲ) stress are randomly selected, primers for these target genes are designed using SnapGene (v 6.1.1), with GAPDH (An07g01150) as the reference gene (primer information provided in Table 3-2), and a 20 μL RT-qPCR reaction system is prepared, including 0.4 μL Forward primer, 0.4 μL Reverse primer, 2 μL cDNA, 10 μL 2×SYBR qPCR Master Mix, and 7.2 μL ddH2O, the mixture is added to a 96-well plate and run on a quantitative real-time PCR instrument with the program of 95 °C for 3 min followed by 45 cycles of 95 °C for 5 s and 60 °C for 30 s.
